# Region-Specific Integration of Embryonic Stem Cell-Derived Neuronal Precursors into a Pre-Existing Neuronal Circuit

**DOI:** 10.1371/journal.pone.0066497

**Published:** 2013-06-20

**Authors:** Franziska Neuser, Martin Polack, Christine Annaheim, Kerry L. Tucker, Martin Korte

**Affiliations:** 1 Zoological Institute, Division of Cellular Neurobiology, TU Braunschweig, Braunschweig, Germany; 2 Biocenter Basel, University of Basel, Basel, Switzerland; 3 Interdisciplinary Center for Neurosciences, Institute of Anatomy and Cell Biology, University of Heidelberg, Heidelberg, Germany; Hertie Institute for Clinical Brain Research, University of Tuebingen., Germany

## Abstract

Enduring reorganization is accepted as a fundamental process of adult neural plasticity. The most dramatic example of this reorganization is the birth and continuously occurring incorporation of new neurons into the pre-existing network of the adult mammalian hippocampus. Based on this phenomenon we transplanted murine embryonic stem (ES)-cell derived neuronal precursors (ESNPs) into murine organotypic hippocampal slice cultures (OHC) and examined their integration. Using a precise quantitative morphological analysis combined with a detailed electrophysiology, we show a region-specific morphological integration of transplanted ESNPs into different subfields of the hippocampal tissue, resulting in pyramidal neuron-like embryonic stem cell-derived neurons (ESNs) in the Cornu Ammonis (CA1 and CA3) and granule neuron-like ESNs in the dentate gyrus (DG), respectively. Subregion specific structural maturation was accompanied by the development of dendritic spines and the generation of excitatory postsynaptic currents (EPSCs). This cell type specific development does not depend upon NMDA-receptor-dependent synaptic transmission. The presented integration approach was further used to determine the cell-autonomous function of the pan-neurotrophin receptor p75 (P75^NTR^), as a possible negative regulator of ESN integration. By this means we used p75^NTR^-deficient ESNPs to study their integration into a WT organotypic environment. We show here that p75^NTR^ is not necessary for integration per se but plays a suppressing role in dendritic development.

## Introduction

Region-specific differentiation and integration of neuronal precursors are crucial for nervous system development. For decades candidates influencing neuronal fate and integration have been discussed. Especially external cues, such as polypeptides secreted by the surrounding tissue or adhesion molecules, and neuronal activity have been known to modulate axonal growth as well as dendritic shape and complexity [1]–[4]. In addition, cell-intrinsic mechanisms, in particular the activation of defined transcription factors, have gained more and more acceptance in defining neuronal fate [5], [6]. However, to what extent these different driving forces go hand in hand and influence one another to determine the final location, subtype identity and dendritic morphology of the respective neuron is currently under debate [7].

Two regions of the rodent brain are capable of adult neurogenesis, the subventricular zone of the lateral ventricle and the dentate gyrus (DG) of the hippocampal formation (reviewed in [8]). The integration of adult-born neurons into the pre-existing neuronal network has been shown on a morphological as well as electrophysiological level (reviewed in [9]) and their unique role in pattern separation during a time window of high excitability is matter of current research [10], [11]. While previous transplantation studies into hippocampal tissue focused on the general integration potential of ESNPs [12], [13], these studies lacked details about quantitative assessment of neuronal activity and morphology, compared to endogenous neurons, of the integrating neurons in the various subfields of the hippocampus. We show here that a population of transplanted ESNPs is capable of morphologically and functionally integrating into the different subfields of the hippocampal formation in culture. For the first time, detailed morphological analysis allows the distinction between ESNs acquiring a pyramidal dendritic tree in the CA regions and ESNs developing granule neuron features in the DG. With respect to the dendritic complexity ESNs integrating into the DG are indistinguishable from resident granule neurons. Thereby the integration process of transplanted ESNPs is most likely independent of classical plasticity-related mechanisms as the activation of NMDA receptors. In order to look therefore for cell-autonomous effects, we used ES cell-derived neuronal precursors lacking the neurotrophin receptor p75 (p75^NTR^). p75^NTR^ is of special interest, since it has on the one hand neurotrophins as ligands and in addition it interacts with Trk-Receptors and even has ligand-independent functions that are of relevance for ESN cell integration [14], [15]. Dependent on the cellular context, the effects of p75^NTR^ range from apoptosis [16], over neuronal survival [17] to long-term depression [18], [19], and negatively modulating dendritic complexity and spine density [20]. And indeed we show here that p75^NTR^ is not necessary for integration per se but plays a growth limiting role in dendritic development.

## Materials and Methods

### Ethic Statement

All procedures were carried out according to the guidelines from Animal Committee on Ethics in the Care and Use of Laboratory animals of TU-Braunschweig and was specifically approved by TU-Braunschweig. The welfare of the mice was ensured according to the guidelines of the Animal Committee on Ethics in the Care and Use of Laboratory animals of TU-Braunschweig. Before animals were sacrificed they are anaesthetized using CO2 anesthesia again in accordance with the Animal Committee on Ethics in the Care and Use of Laboratory animals of TU-Braunschweig.

### Materials

Cell culture media and solutions were purchased from Gibco (life technologies, Carlsbad, USA) and chemicals from Sigma (St. Louis, USA) unless otherwise stated. The batches of fetal calf serum (FCS) used for ES cell culture were especially tested for the differentiation procedure. Leukemia inhibitory factor (LIF) was produced in COS-7 cells that were chemically transfected with a LIF over-expressing plasmid. The harvested medium was frozen in aliquots and confirmed to inhibit unspecific differentiation of ES cells.

### Differentiation of ES Cells into Neuronal Progenitors (ESNPs)

Differentiation of mouse ES cells was performed as described by Bibel et al. [21], [22]. Briefly, ES cells in LIF-containing medium were passaged before reaching confluence, until a mouse embryonic fibroblast (MEF)-free culture was obtained. 3.5×10^6^ cells were sowed onto bacteriological dishes (Greiner) in 15 ml EB medium, where they formed cellular aggregates, so-called embryoid bodies (EBs). The medium was changed after 2, 4 and 6 d, respectively, while at 4 and 6 d 5 µM retinoic acid was added. After 8 d in culture, EBs were washed in PBS and subsequently dissociated using freshly thawed trypsin (0.05% in 0.04% EDTA in PBS), followed by a shaking incubation at 37°C for 3 min. The reaction was stopped by adding 10 ml of EB medium and re-suspension. Following centrifugation, the pellet was adsorbed in EB medium, and cells were filtered through a 40 µm cell strainer (BD Falcon) to remove residual cell clumps. 2× cryo medium was added to freeze aliquots of ESNPs.

### Preparation of Organotypic Hippocampal Cultures (OHCs)

Cultures were prepared and cultivated as previously described [23] from postnatal day 5 (p5) wild type mice (C57Bl/6). Briefly, the hippocampi were isolated and transferred into cooled Gey’s balanced salt solution (GBSS) containing 0.5 ml of kynurenic acid (100 mM stock solution) and 0.5 ml of glucose (50% stock solution), adjusted to pH 7.2. Using a McIllwaine tissue chopper (Wood Dale, IL), the hippocampi were cut into 400 µm slices. The slices were incubated in cooled GBSS for 30 min at 4°C before being placed onto Millicell® cell culture inserts (Millipore, Bedford, MA) in 6-well plates containing culture medium (50% DMEM with HBSS without glutamine, 25% HBSS, 1 ml of glucose (50%), 25% Donor Equine Serum (HyClone, Logan, UT), and 0.5 ml of L-glutamine (200 mM stock solution). After 72 h of cultivation at 36.5°C and 5% CO_2_, a mixture of antimitotics (cytosine arabinoside, uridine, and fluorodeoxyuridine, 10^6^ to 10^7^ M each) was added to the wells. 24 h later, the inserts were transferred into a new plate containing pre-warmed culture medium. Slices were cultured for up to six weeks, the medium being changed twice a week, with 1.25 µg/ml Fungizone (Gibco) as well as 100 U Penicillin and 100 µg/ml Streptomycin (PAA) being added to the fresh medium.

### Particle-mediated Gene Transfer

Intrinsic hippocampal neurons were transfected with a plasmid containing farnesylated EGFP, using biolistic particle-mediated transfer via the Helios Gene Gun (Bio-Rad, Hercules, CA). First, 600 nm gold micro particles were loaded with the plasmid according to the instruction manual for the Helios Gene Gun (Bio-Rad). The Tefzel tube was inserted into the tubing preparation station (Bio-Rad) and blown with nitrogen for 10 min before the gold suspension was loaded. After letting the gold sink for 3–5 min, the ethanol was removed. For a homogenous distribution of the gold particles, the tube was rotated for 30 s, followed by a drying period of 5 min. Shooting was done using helium pressure (100 psi) and a 3 µm filter (BD Falcon) in front of the barrel aligner to prevent damage of the culture by gold clusters.

### Transplantation of ESNPs

Glass micropipettes were produced using a vertical puller (Narishige, Japan) and glass capillaries (1.5 mm O.D. × 0.86 mm I.D., Harvard Apparatus, Holliston, USA). The “step2 heater” setting of the puller was used and the capillary was stretched for 8 mm between the two heating steps in order to generate a needle opening size between 25 and 35 µm. The frozen progenitors were centrifuged for 5 min in 20 ml EB medium, the pellet was re-suspended in 100 µl N2 medium [22], and the cell suspension was transferred into a suspension culture dish to be stored inside the incubator for up to 30 min. ESNPs were transferred into the pipette using a syringe (Braun, Melsungen, Germany). OHCs at 5 d after preparation were transplanted with ESNPs, using a micro injection device (Picospritzer, Toohey Company, New Jersey, USA) with 10 psi and a 0.1 ms injection pulse. A micromanipulator (Leitz, Wetzlar, Germany) in combination with an electrode holder (Biomedical Instruments, Zöllnitz, Germany) were used to position the micropipette within the culture. Upon transplantation, the tissue of the main regions of the hippocampus, CA1, CA3 as well as the dentate gyrus (DG) was penetrated and the progenitors were injected into the cell body layers. Based on the injection volume of 0.02 µl and a viability of about 5% of the frozen progenitors (estimated by plating on glass cover slips and comparing to embryonic hippocampal neurons) the number of transplanted viable ESNPs was 50 per slice. At DIV 32 on average 4.3 ESNs survived in the slice culture (Fig. S1).

### Electrophysiology

OHCs were prepared and transplanted as described above. Whole-cell patch clamp recordings were obtained from ES cell-derived neurons (ESNs) and hippocampal pyramidal neurons from OHCs at room temperature and 32°C at 28–35 DIV. Patch-clamp electrodes were pulled from borosilicate glass (Harvard-Apparatus, Holliston, USA) using a vertical puller (Narishige, Japan) to a resistance of 5–8 MΩ. For experiments in which the holding potential was −40 mV the following pipette solution was used (in mM): CsCl, 140; CaCl2, 1; MgCl2, 2; HEPES, 10; EGTA, 11; Na-ATP, 2. All other experiments: K-Gluconate, 120; KCl, 17,5; NaCl, 10; MgCl2, 3; CaCl2, 1; HEPES, 10; EGTA, 0,5; Na-ATP, 2. Both solutions were adjusted to pH 7,2 with either CsOH or KOH and 290 mOsmol/kg and contained Alexa 568 (Invitrogen) for labeling. Miniature EPSCs were pharmacologically isolated by adding TTX (1 µM, Tocris, Ellisville, USA) to the bath solution (in mM): NaCl, 125; KCl, 2,5; NaH2PO4, 1,25; MgCl2, 1; CaCl2, 2; NaHCO3, 26; Glucose, 25; pH 7,4. D-AP5 (50 mM, Tocris), the GABA_A_-receptor blocker picrotoxin (50 µM, Tocris) and CNQX (50 mM, Tocris) were used in pharmacological experiments. After obtaining a stable gigaseal (>1 GΩ) recordings were made using an Axopatch 200B (Axon Instruments, Foster City, USA) amplifier. Series resistance was monitored and compensated to 85% and experiments were rejected if 50% or 25 MΩ were exceeded. Signals were filtered using a 5 kHz Bessel filter and digitized at 20 kHz using a Digidata 1322A (Axon Instruments). Signals were acquired and analyzed with pClamp 9 (Axon Instruments).

Target cells were detected by either fluorescence- or differential interference contrast microscopy using an upright Axioskop 2 FS plus microscope (Zeiss, Germany). Cells were patched in the CA1 or CA3 region of the hippocampus and in some experiments additionally stimulated via the mossy-fiber pathway or Schaffer collaterals by placing a monopolar tungsten electrode (WPI, 10 MΩ) in the dentate gyrus or CA3 region of the OHC. For isolating receptor mediated currents EPSCs were recorded with the following protocol: 10 min at −65 mV, 10 min at −40 mV, 10 min with D-AP5, 10 min with D-AP5 and CNQX followed by 20 minutes washout. To examine mEPSCs neurons were kept under voltage-clamp over 10–20 min at a holding potential of −65 mV. Action-potential- and spiking properties were examined by current-clamp recordings. Cells were step depolarized and hyperpolarized for 1 s with 200 pA. All ESNs were identified by EGFP epifluorescence and directly checked after the experiment for co-staining of EGFP and Alexa 568.

### Immunohistochemistry

After transplantation of ESNPs, immunohistochemistry using a αGFP antibody was performed on a routine basis in order to increase the EGFP signal. OHCs were fixed over night in freshly thawed 4% PFA solution in 0.1 M phosphate buffer at 4°C. The cultures were washed eight times with phosphate buffered saline (PBS) for 15 min and quenched in 50 mM NH_4_Cl in PBS for one hour. Subsequently, the OHCs were incubated with blocking solution (1% bovine serum albumin (BSA), 10% goat serum (PAA), 0.2% Triton-X-100) over night at 4°C. GFP-monoclonal (Millipore MAB3580, 1∶500), GFP-polyclonal (Millipore AB3080, 1∶500), synapsin1 (Millipore AB1543, 1∶500) and vGLUT1 (SYSY 135311, 1∶5000) antibodies were used as primary antibodies and incubated in blocking solution without BSA for 6 d at 4°C. Before incubating with the secondary antibodies (Cy2/Cy3-conjugated, Jackson Immuno Research, Philadelphia, USA) in PBS for 2 d, the slices were washed eight times for 15 min in PBS. Finally, the cultures were stained with DAPI (1∶1000 in PBS) and mounted with Fluoro-Gel®.

### Neuronal Imaging and Analysis

During the culture period, ESNs growing in OHCs were pictured repeatedly, either to localize interesting neurons that would be matter of investigation later on, or to perform time-lapse imaging (using an Olympus BX61WI microscope equipped with a 10×, 20× and a 40× objectives combined with the Cell∧M software). Live microscopy was also used to image dendritic spines. Culture inserts were transferred into a 35 mm dish containing a drop of pre-warmed Hank’s balanced salt solution (1× HBSS supplemented with 4.17 mM NaHCO_3_ and 2 mM CaCl_2_) and covered by 2 ml additional HBSS. Beyond that, live imaging was performed as previously described [24], [25]. When screening the slices, single plane records were taken to recognize a specific ESN later on. While screening after 28 to 32 days *in vitro* (DIV), ESNs were attributed to the different ESN types A and B (see below). For analysis of the three-dimensional structure, image stacks were recorded, the step width being dependent on the chosen objective.

Following a culture period of 32 DIV, slice cultures were fixed and immunohistochemically stained for EGFP. Within the mounted slices, neurons were imaged at the Axioplan2 microscope combined with an ApoTome module (Zeiss, Jena, Germany), using a 20× objective and 1 µm step width to capture their entire dendritic tree. Image stacks were transferred into the Neurolucida® software and traced (MicroBrightField, Colchester, VT). In case following the dendritic tree revealed a secondary cell body-like swelling, tracing was stopped when this structure was reached (Fig. S2D). Sholl analysis was performed in order to determine dendritic length and complexity [26], separately for basal and apical dendrites. We also calculated the total number of crossings as an index for total dendritic complexity. Mean values were determined for the neuron population, and the standard deviation of the mean was divided by the square root of the analyzed neuron number to calculate the error. Synapses were detected by co-staining fixed slice cultures with antibodies against EGFP and a presynaptic marker. Three dimensional stacks of multi-channel images were taken at the LSM5-Meta confocal laser scanning microscope (Zeiss, Jena, Germany) to co-localize EGFP (amplified by αGFP-Cy2) as well as Cy3 signal at the synapses. A 40× water immersion objective was used, combined with a digital 4× zoom and a step width of 0.3 µm. An EGFP-positive structure overlapping with or directly neighbored by a Cy3-positive dot in the same focal plane, one below or one above, was considered a synapse.

### Statistical Analysis

Statistical significance was determined by the two-tailed Student’s t test (type 3) or by using a two-way ANOVA followed by a Bonferroni post test. All data are shown as the mean ± SEM. Statistical significance was presumed when p<0.05. In figures, *p<0.05, **p<0.01 and ***p<0.001.

### Classification of ESNs

The quantification of type A (morphologically hippocampal-like) and type B (morphologically immature) ESNs was done using live microscopy. Slices were screened and cells meeting the following criteria were attributed to type A ESNs: 1) Soma position within the intrinsic cell body layer, 2) Complex and defined dendritic tree, pyramidal cells composed of basal and apical compartment, 3) Orientation according to the intrinsic cytoarchitecture, 4) Pyramidal neurons: Apical dendrite longer than basal ones. All cells that had to be excluded from type A ESNs based on these criteria were considered type B ESNs. After fixation and immunohistochemistry, compliance with the aforementioned criteria was critically confirmed before attributing an ESN to type A ESNs for the morphological analysis. Because of their less complex dendritic tree, granule-like type A ESNs could only be specified after fixation making it impossible to record from DG ESNs.

### Cell Lines

Both the wildtype ES cell line, designated M22, and the p75^NTR^-KO cell line, designated B7–15, used a 7.6-kb fragment of the murine tau (*Mapt*) promoter, which endogenously controls transcription of the *Mapt* gene [27], to drive EGFP expression. A Pgk-NEO^R^ cassette allowed for selection of positive ES cell clones, and the construct inserted with multiple copies randomly into the genome. The homozygous p75^NTR^-KO cell line was produced by sequential introduction of a floxed *exonIV* cassette followed by *cre* recombination.

## Results

### Distinct Morphological Types of ES Cell-derived Neurons

**Figure 1:**
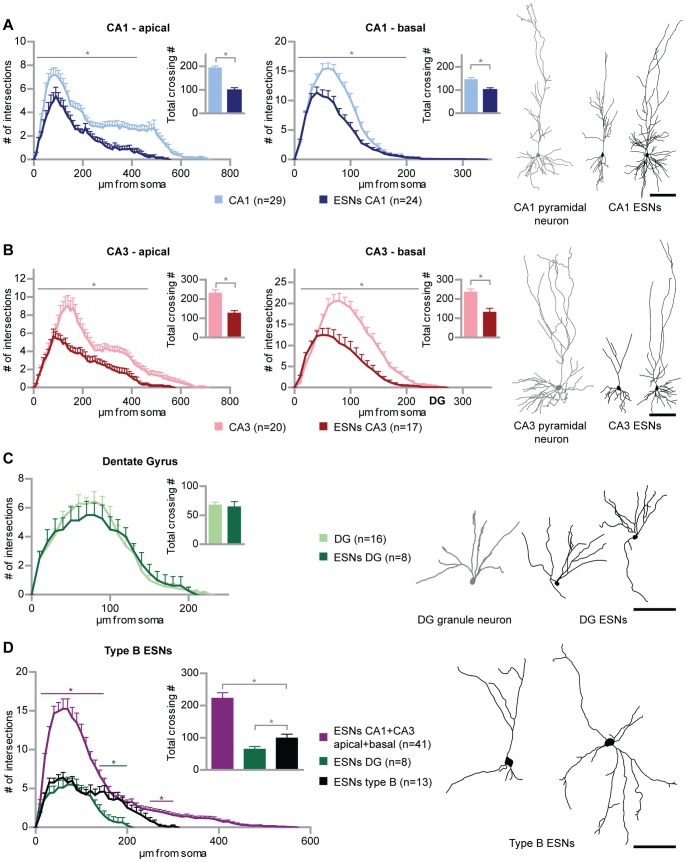
Generation of morphologically different embryonic stem cell-derived neurons (ESN). (A) ESNs in the CA1 region reproduce the morphology typical of intrinsic CA1 pyramidal cells. Sholl analysis of ESNs in the CA1 region at 21–32 DIV (dark blue) compared to EGFP-f transfected hippocampal neurons at 25 DIV (light blue) for apical and basal dendrites, respectively. (B) CA3 ESNs (dark red) morphologically resemble intrinsic CA3 pyramidal cells (light red) as shown via Sholl analysis and tracing examples. (C) ESNs in the DG (dark green) reproduce the morphology and orientation of intrinsic granule neurons (light green; transfected at 11 and analyzed at 14 DIV) and show the same degree of complexity. (D) Morphologically immature ESNs – type B ESNs – significantly differ from the ESN populations described in (A) to (C) in length and complexity. Numbers of intersections of apical and basal dendrites were totaled in order to compare the different ESN morphologies. Colored significance bars refer to type B ESNs versus ESNs in CA1 and CA3 (purple) and type B ESNs versus ESNs in the DG (green), respectively. For the sake of clarity, significance bars for ESNs in CA1 and CA3 versus ESNs in the DG have been omitted. Student’s t test, *p<0.05. Error bars represent SEM; Scale bars represent 100 µm.

Initially, we examined whether pre-differentiated embryonic stem cell-derived neuronal progenitors (ESNPs) are capable of morphologically integrating into organotypic hippocampal slice cultures (OHCs). ES cells carrying a *tau*EGFP transgene were differentiated into neuronal progenitors as described previously [21], [22]. This differentiation procedure has been designed to yield a neuronal progenitor population highly enriched of Pax6-positive radial glial cells, which are known to represent the progenitors of cortical and hippocampal pyramidal neurons [21]. In the present study, the differentiation process was followed by transplantation of the obtained ESNPs into OHCs prepared from P5 wild type mice. As a result the generation of morphologically different cell types was observed. The focus of the present study was to investigate the resulting ES cell-derived neurons (ESNs), although in some cases also smaller cells – resembling glia - were found in the OHCs (Fig. S3A,D). The obtained ESNs profoundly differed in size and shape, revealing two structurally different ESN groups. While the larger group (96.1% of 1036 ESNs in 240 cultures) displayed an undirected dendritic growth and were found anywhere in the OHC unrelated to the intrinsic hippocampal structure, a smaller fraction of transplanted ESNPs (3.9%) adopted hippocampal cell morphologies (Fig. 1; for an overview of a transplanted OHC and original micrographs of CA1, CA3 and DG ESNs see also Fig. S2). This structurally hippocampal-like fraction of ESNs was exclusively located within the intrinsic cell body layers of the respective hippocampal subfields (49 ESNs were analyzed in total). Based on precise criteria (see Materials and Methods), ESNs resembling intrinsic hippocampal neurons and adopting their typical orientation (“type A ESNs”, Fig. 1A–C) were distinguished from the morphologically immature remainder (“type B ESNs”, Fig. 1D). Type A ESNs developed distinct morphological features according to the hippocampal region they were located in (CA1, CA3 region and DG). We used Sholl analysis to compare the dendritic length and complexity of ESNs at 32 days *in vitro* (DIV) post transplantation, to intrinsic hippocampal neurons that had been transfected with a farnesylated form of EGFP (EGFP-f) via particle-mediated gene transfer (transfected at 21 DIV, fixed at 25 DIV; intrinsic hippocampal neurons of this age are considered mature; however we kept ESNs in culture up to 32–35 DIV in order to compare mature ESNs to mature intrinsic neurons and thus to compensate for a potentially delayed development as described for adult-generated granule neurons [28]). The analysis revealed that the characteristic morphology of intrinsic hippocampal neurons was reproduced by type A ESNs found in the CA1 and CA3 region, respectively (Fig. 1 A,B). Nevertheless, the dendrites of ESNs in the CA1 and in the CA3 region were shorter and significantly less complex than their resident hippocampal counterparts. This applied to both the apical and to the basal dendritic compartment (total dendritic complexity, ESN vs endogenous pyramidal neurons: CA1 apical p<0.001; CA1 basal p = 0.0014; CA3 apical p<0.001; CA3 basal p<0.001). Comparing pyramidal-like CA1 and CA3 ESN morphology to type B ESNs (live after 28 DIV), revealed highly significant differences between the two groups (Fig. 1D; p<0.001). Type B ESNs were dramatically less complex and shorter than pyramidal type A ESNs. Even after repeatedly imaging 50 type B ESNs over time from 2 to 29 days in culture, no type B ESN was found to develop into a type A ESNs.

### ES Cell-derived Neurons Reproduce Hippocampal Neuron Morphology in a Region-specific Manner

Similarly, progenitors were transplanted into the dentate gyrus (DG), and surviving ESNs were detected, some of which were considered to be type A ESNs (according to the criteria, see Materials and Methods) and thus included into the morphological analysis (Fig. 1C; see also Fig. S2B). In contrast to pyramidal-like ESNs which were less complex and shorter than their intrinsic counterparts, granule-like ESNs not only reproduced the structure but also the length and the degree of complexity of intrinsic granule neurons. According to the selected tracings, they were indistinguishable. Sholl analysis and total complexity also reflected this tendency with only minor differences (around 60 µm to 100 µm from the soma) that were not statistically significant (p>0.05). Thus, ESNPs were capable of morphologically developing into granule cells when injected into the DG. By performing Sholl analysis type B ESNs turned out to be longer and significantly more complex in the distal part of the dendritic tree, resulting in a significant difference in the total crossing number, as well (Fig. 1D; p = 0.03). Thus, the existence of morphologically differing ESN types – type B, pyramidal type A and granule type A – could be confirmed.

**Figure 2:**
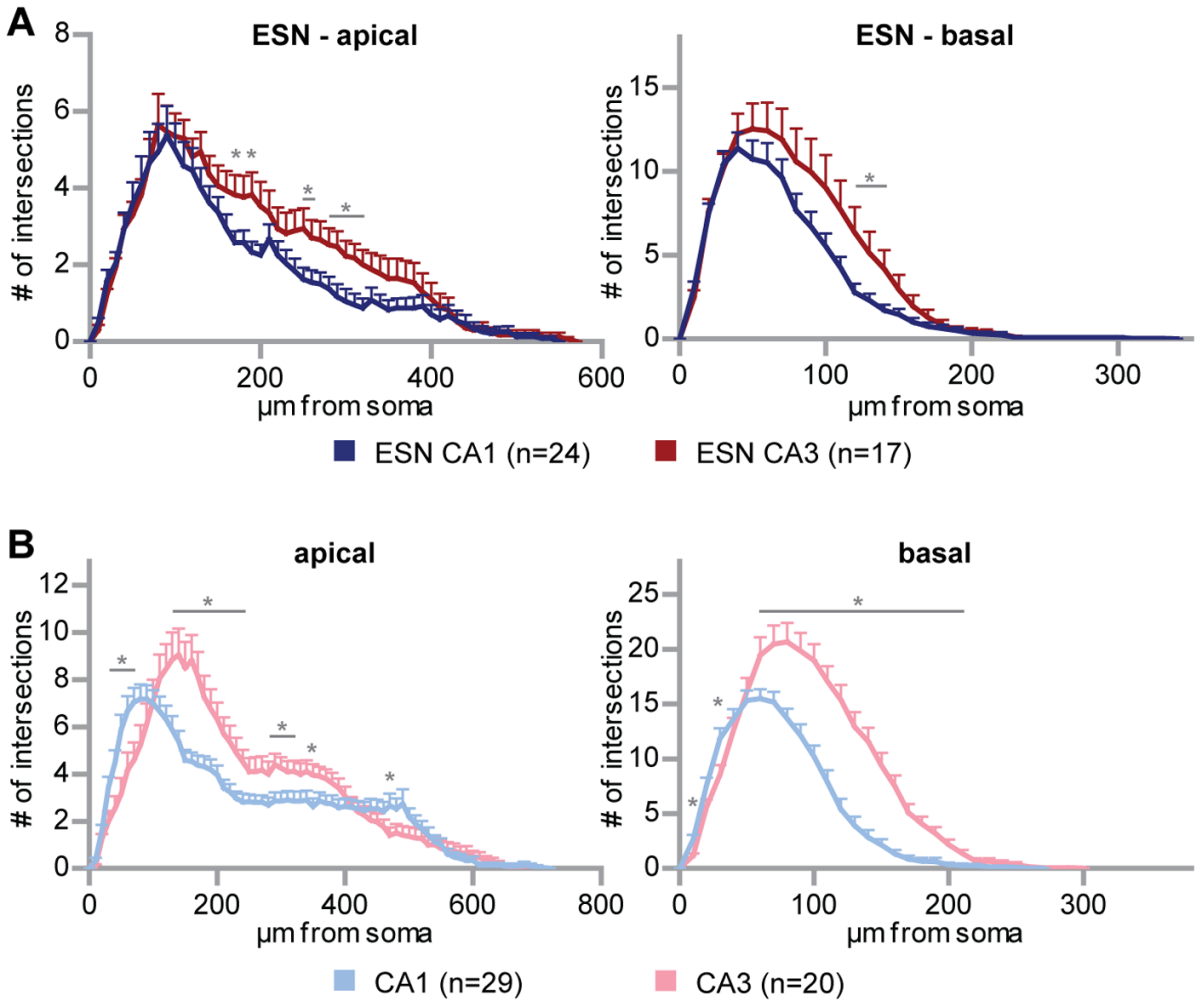
Region-specific generation of pyramidal-like ES cell-derived neurons. (A) Sholl analysis of CA1 versus CA3 ESNs. Note that the number of intersections of CA1 and CA3 pyramidal ESNs differs significantly at distinct distances from the soma. (B) Sholl analysis of intrinsic CA1 versus CA3 pyramidal neurons, each showing a specific curve. Student’s t test, *p<0.05; error bars represent SEM.

Subsequently, we analyzed whether the ESNs in CA1 and CA3 regions produced one uniform pyramidal phenotype or if they were able to react to local information and express characteristic features of the neurons in the two distinct CA subdivisions. For this purpose the complexity curves of both, CA1 and CA3 ESNs were plotted against one another to reveal whether they actually represented two distinct populations. Intrinsic pyramidal CA1 and CA3 neurons showed, as expected, two significantly different curves for the apical and for the basal compartment, respectively (Fig. 2B; e.g. for apical dendrites at 30–70, 130–240, 280–320, 350, 470 µm from soma p<0.05; for basal dendrites at 10, 30, 60–210 µm from soma p<0.05), CA3 neurons being more complex in most regions of the dendritic tree. Comparing both ESN graphs, the proximal part of the tree shows overall the same degree of complexity (Fig. 2A). Following the dendritic tree, however, statistically significant differences between both neuronal groups existed. This applied to the apical (at 170, 190, 250–260, 280–320 µm from soma p<0.05) as well as the basal dendrites (at 120–140 µm from soma p<0.05), and revealed a higher complexity for CA3 ESNs making the results comparable to the endogenous CA3 neurons. In total, the comparison led to the conclusion that CA1 ESNs indeed differed from CA3 ESNs in terms of their dendritic complexity pattern, in a way that is comparable to original hippocampal pyramidal cells. Therefore, the generation of the three principal hippocampal neuron types – granule neurons, CA1 and CA3 pyramidal cells – from a homogenous group of ESNPs could be shown.

### Synaptic Integration of ES Cell-derived Hippocampal-like Neurons

**Figure 3:**
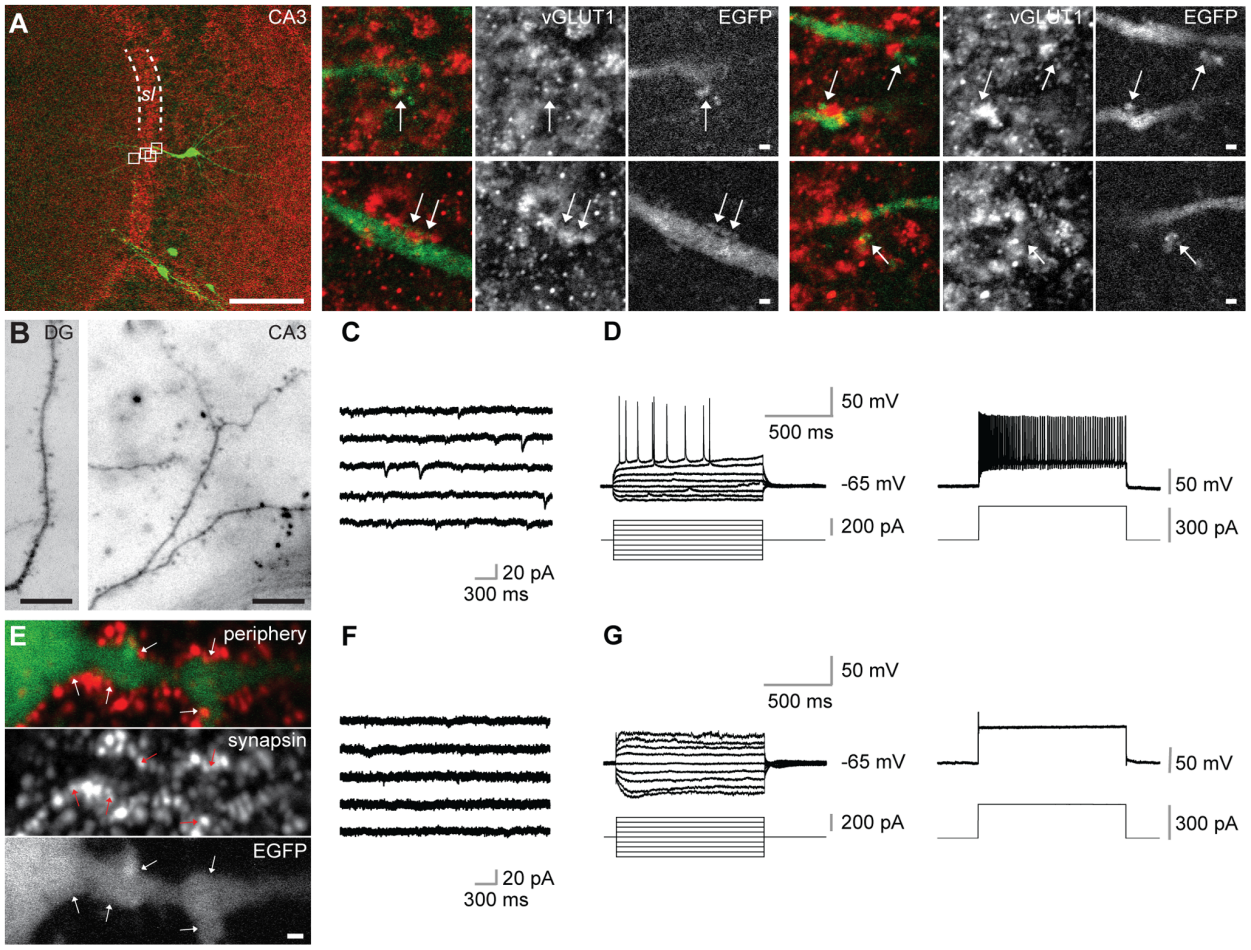
Type A ESNs carry dendritic spines, show EPSCs and fire action potentials. (A) Co-immunohistochemistry against EGFP and vGLUT1 of type A ESN in the CA3 region of an organotypic hippocampal culture. Detailed images at the right side represent magnification of the four white boxes in the left overview. Arrows point to co-localizations of both markers (*sl* = *stratum lucidum*). (B) Type A ESNs in different hippocampal subfields carrying spines. (C) Representative miniature EPSCs of type A ESN in the CA3 region of an OHC. (D) Current-clamp recording from a type A ESN in the CA3 region shows action potentials at distinct levels of depolarization by current pulses (left) and high-frequency firing (right) during a strong depolarization. (E) Type B ESNs are morphologically immature, they do not carry spines. Nevertheless, their dendrites co-localize with the pre-synaptic marker synapsin. ESN found in the periphery of the OHC at 24 DIV. (F) Representative recording showing absence of mEPSCs in type B ESN in the CA3 region. (G) Current-clamp recording showing disability of type B ESN in CA3 region of firing action potentials (left) even during strong depolarization (right). Scale bars in (A) 100 µm (overview) and 1 µm (detailed images), respectively; in (B) 10 µm; (E) 1 µm.

We further investigated whether ESNs were capable of receiving synaptic input. Dendritic spines are generally regarded as a morphological prerequisite for mature excitatory synapses. The dendrites of type A ESNs clearly carried dendritic spines (Fig. 3A, B; n = 9 ESNs from 9 cultures in which spines were unambiguously detected), which were found on ESNs in the three examined regions of the hippocampal DG (Fig. 3B), CA1 (not shown) and CA3 (A, B). Interestingly, spines were most often seen on CA3 pyramidal ESNs. According to the intrinsic cytoarchitecture, the apical dendrite of these ESNs extends toward the centre of the slice crossing the *stratum lucidum,* a region which is heavily innervated by mossy fiber terminals (Fig. 3A). Within the *stratum lucidum*, large and partially branched spines were detected on the dendrites of ESNs, which co-localized with vGLUT1-positive structures (Fig. 3A), suggesting that CA3 ESNs receive mossy fiber input from host granule neurons. Imaging dendrites of CA3 ESNs at different time points revealed that spine growth was age-dependent: While at 14 and 21 DIV spines were never seen (not shown), spiny dendrites were present in ESNs at 28 and 32 DIV (Fig. 3A, B). In contrast to this, dendrites of type B ESNs showed a less well defined dendritic fine architecture devoid of typical spines (consisting of neck and head, Fig. 3E). Our observations were supported by patch-clamp recordings of type A and B ESNs. The absence of mEPSCs and characteristic action potentials in type B ESNs (Fig. 3F,G) revealed that only type A cells have the ability for spontaneous synaptic currents (Fig. 3C) and action potential firing (Fig. 3D).

### Type A ESN EPSCs and AP-firing Properties are Comparable to Intrinsic Hippocampal Neurons

**Figure 4:**
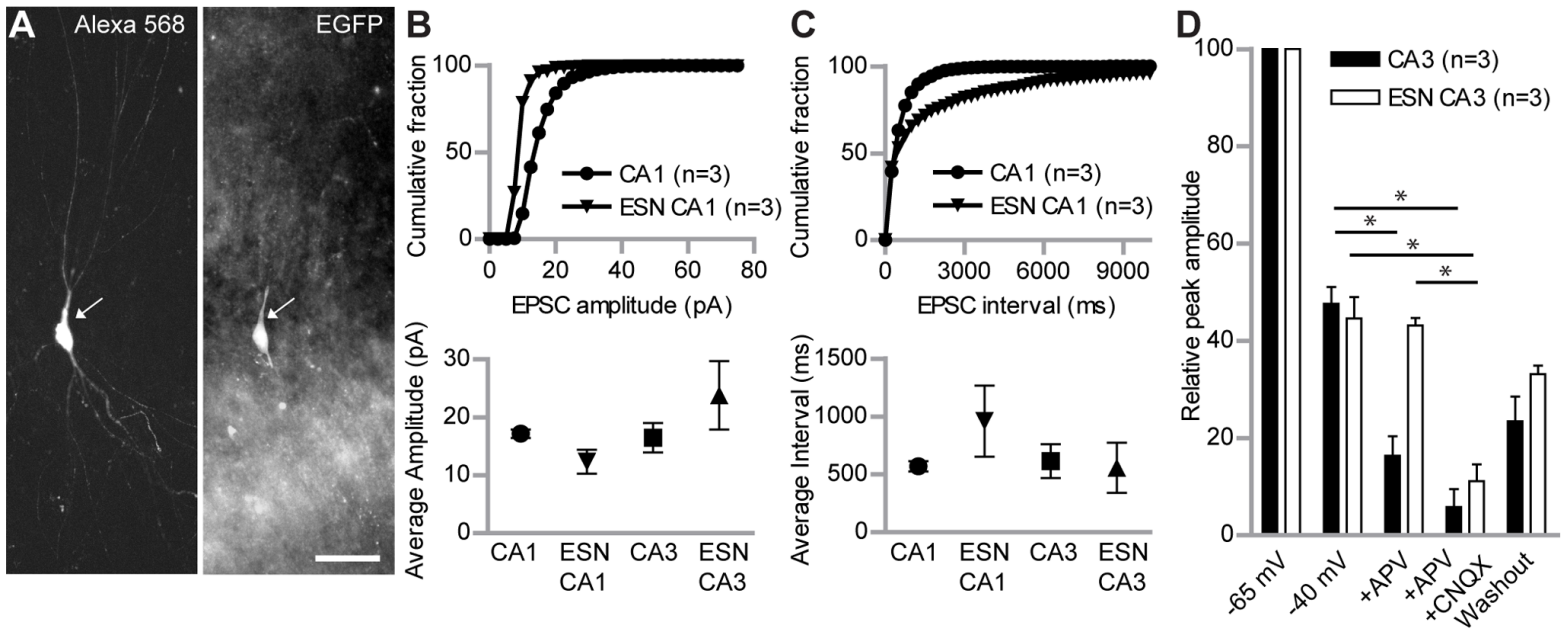
Synaptic input properties of type A ESNs versus control neurons. (A) Type A ESN in the CA3 region that is filled with Alexa 568 during patch-clamp recording (left); the same cell is EGFP-positive (right). (B) Cumulative percentage of mEPSC amplitudes recorded in voltage-clamp recordings of WT neurons and ESNs in the CA1 region. The curve of ESNs reaches 100% faster, indicating more mEPSCs with lower amplitude than in WT neurons (top). This tendency is supported by the total average amplitude in CA1 but not CA3 neurons (bottom). (C) Cumulative percentage of mEPSC intervals of type A ESNs and WT neurons recorded in voltage-clamp experiments. The curve of CA1 ESNs reaches 100% later, indicating longer intervals between mEPSCs, compared to WT neurons (top). The total average of mEPSC intervals shows no significant difference for both CA1 and CA3 ESNs (bottom). Number of underlying neurons in (B) and (C) - CA1: n = 3; ESN CA1: n = 3; CA3: n = 6; ESN CA3: n = 4. (D) Comparison of relative peak amplitudes of distant stimulation-evoked EPSCs in type A ESNs and WT neurons in the CA3 region under different recording conditions. WT neurons show significantly lower amplitude after application of APV and an almost complete block after application of APV/CNQX. Conversely, ESNs show no change in amplitude after APV application, but their amplitude is significantly reduced after APV/CNQX application, the value being indistinguishable from WT neurons. Student’s t test, *p<0.05. Error bars represent SEM; Scale bar represents 50 µm.

For further physiological investigations we focused on type A ESNs in the CA1 and CA3 region of the hippocampus. To ensure that patch-clamp recordings were only performed from ESNs, the EGFP positive ESNs were filled with Alexa 568 during all experiments via the patch pipette and afterwards checked for co-localization of both signals (Fig. 4A). Stable voltage-clamp recordings were obtained to characterize the electrophysiological properties of cells at 26–34 DIV after transplantation. All investigated cells showed action potentials that were sensitive to the application of tetrodotoxin (TTX, 1 mM). The average amplitude of mEPSCs was not significantly different between ESNs and intrinsic neurons in the corresponding region (Fig. 4B, bottom). CA1 ESNs showed a tendency to smaller mEPSC amplitudes in the cumulative fraction (Fig. 4B, top). In line with these results the average interval between the mEPSCs was not significantly different between ESNs and control neurons (Fig. 4C bottom), while the cumulative fraction of ESNs in the CA1 region showed a tendency to longer intervals (Fig. 4C, top).

**Figure 5:**
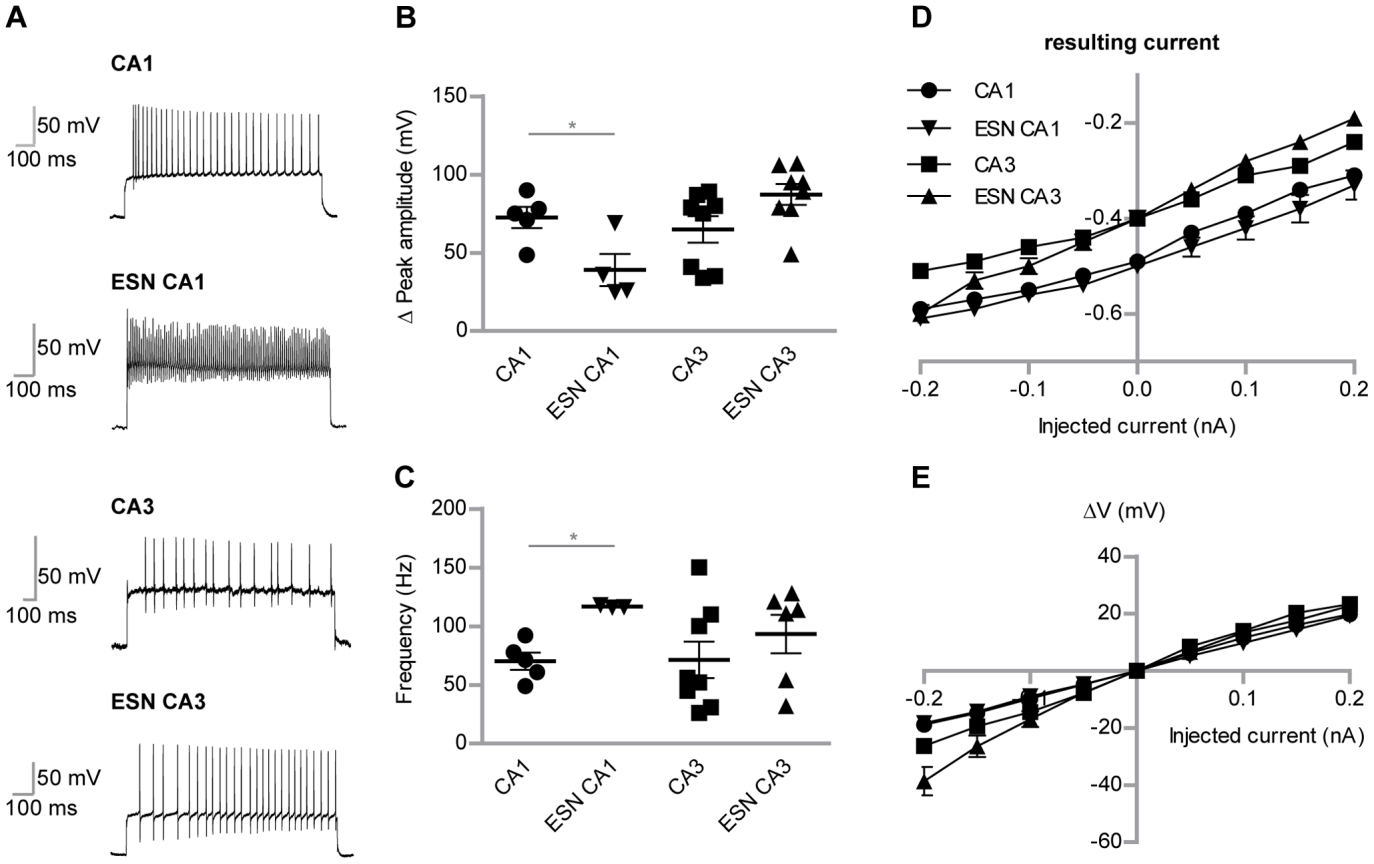
Detailed comparison of AP-firing potentials of ESNs and control neurons. (A) Peak amplitudes of action potentials during depolarization in current-clamp recordings. Type A ESNs in CA1 region show significantly lower peak amplitudes compared to WT neurons, while the frequency (B) is significantly higher. (C) Representative single traces of action potential frequencies. (D) Injected versus resulting currents plotted for both WT neurons and ESNs in CA1 and CA3 regions, respectively. Depending on their spatial integration, ESNs show resulting currents comparable to WT neurons. (E) The I-V curve shows the peak-current for WT neurons and ESNs in CA1 and CA3 regions. Student’s t test, *p<0.05. Error bars represent SEM.

As one of the main indicators for (mature) neurons we next investigated action potential properties. Action potentials were enforced by giving a square pulse of 300 µA for 1 sec in current-clamp mode (Fig. 5A). While CA3 ESNs showed comparable characteristics for peak amplitudes (Fig. 5B) and frequencies (Fig. 5C) of action potentials to CA3 control neurons, CA1 ESNs produced significantly smaller amplitudes and higher frequencies (p<0.05). Current-voltage characteristics were examined by applying depolarizing and polarizing currents (Fig. 5D, E). ESNs showed a tendency to region-specific resulting currents but no difference in membrane potential-depending changes during current stimuli (Fig. 5E). Passive membrane properties were within the same range, too, except for the capacitance, where higher values were observed (Table 1). The input resistance of mature ESN CA1 neurons was not significantly different to endogenous neurons: 475.1±41.3 MΩ (CA1, endogenous control neurons) vs 441.7±33.8 MΩ (CA1, ESN neurons). This indicates that indeed the ESN neurons resemble mature neuronal properties.

**Table 1:** Comparison of capacitance (Cap.), resting membrane potential (RMP) and action potential (AP) threshold of resident hippocampal neurons, wild type and p75^NTR^-KO ESNs.

	CA1	ESN CA1	P75 ^NTR^-KO CA1	CA3	ESN CA3	P75 ^NTR^-KO CA3
**Cap. (pF)**	152.1±19.6	297.3±15.8	288.7±44.4	48.2±6.5	235.1±19.0	239.7±28.9
***n***	11	13	3	3	3	3
**RMP (mV)**	−65.4±3.3	−53.3±6.0	−51.5±1.3	−67.8±1.0	−67.5±2.1	−49.3±1.7
***n***	5	6	4	5	3	4
**AP Threshold (mV)**	−29.0±1.2	−28.0±4.2	−38.3±0.9	−31.4±1.2	−38.5±2.7	−39.5±0.7
***n***	5	4	4	5	3	4

### Properties of CA3 ESN EPSCs

In addition to the intrinsic electrical properties we tested the connectivity of ESNs in the CA3 region to the pre-existing hippocampal network. A stimulating electrode was placed into the dentate gyrus while ESNs in the CA3 region were patched in voltage-clamp mode. Additionally, APV and CNQX were used to discriminate between different AMPA receptor and NMDA receptor-mediated currents in the ESN. All experiments were performed in the presence of picrotoxin (50 µM), in order to avoid outward synaptic GABA_A_ receptor-mediated currents. The relative peak amplitude of EPSCs at −65 mV and −40 mV was comparable in ESNs and intrinsic control neurons. But whereas intrinsic CA3 pyramidal cells were sensitive to the blockade of the NMDAR blocker APV (50 µM), EPSCs from ESNs did not change after APV application (Fig. 4D). On the other hand all signals from intrinsic CA3 pyramidal cells and from ESNs were completely blocked by APV and CNQX, indicating that ESNs show currents mediated by AMPA receptors but we did not observe NMDA receptor mediated currents. These results led us to ask, if for transplanted ESNPs intrinsic factors together with extracellular, region-specific cues, lead to different hippocampal neuronal cell types.

### Type A ESNs Lacking p75^NTR^ are more Complex and Show a Higher EPSC Amplitude

**Figure 6:**
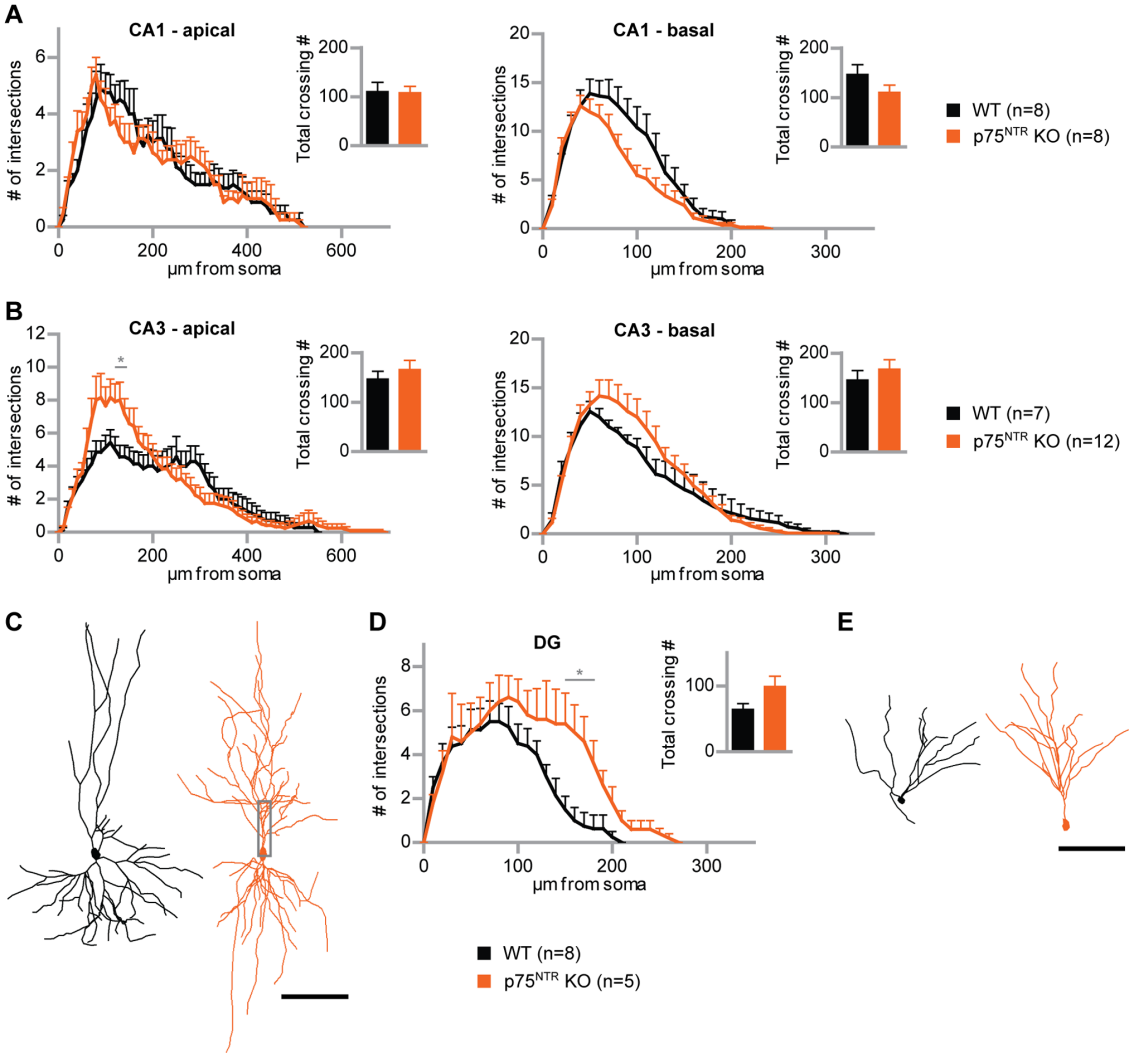
p75^NTR^-deficient type A ESNs are more complex than the WT. (A) Sholl analysis of p75^NTR^-deficient pyramidal-like ESNs (orange) compared to WT ESNs (black) in the CA1 region, for apical and basal dendrites, respectively. (B) Sholl analysis of p75^NTR^-knockout ESNs versus WT ESNs in CA3. (C) Tracing examples of WT and p75^NTR^-knockout ESNs in CA3. (D) Sholl analysis of granule cell-like ESNs in the DG, p75^NTR^-deficient and WT, respectively. P75^NTR^-knockout ESN dendritic trees are longer and significantly more complex. (E) Tracing examples of granule cell-like ESNs in the DG, WT versus p75^NTR^-knockout. Student’s t test, *p<0.05; error bars represent SEM. Scale bars 100 µm.

According to the method described above in Bibel et al. [22], p75^NTR^-KO ES cells were differentiated into neuronal progenitors. The used knockout ES cell line – which was homozygous for *exonIV* of the p75^NTR^ gene – carried multiple copies of the same *tau*EGFP transgene as the wild type line that was used in this study. Based on the known role of p75^NTR^ in shaping neuronal morphology in mature OHCs [20] we examined whether the lack of p75^NTR^ has a cell-autonomous impact on the dendritic complexity of hippocampal-like (type A) ESNs. To this end, p75^NTR-^KO ESNPs were transplanted into OHCs, incubated for 32 days and fixed afterwards. As described above for the wild type, a wide range of neuronal morphologies was found revealing the same outcome in ESN types as for WT ESNs. Populations of pyramidal- and granule-like ESNs (type A ESNs) lacking p75^NTR^ were compared to the respective populations of wild type ESNs, which were found in the same hippocampal regions (Fig. 6; see Fig. S4 for original micrographs of p75^NTR^-KO ESNs). In case of CA3 ESNs, the p75^NTR^-KO cells (n = 12 p75^NTR^-KO neurons from 10 cultures) showed an increase in complexity compared to the wild type control (n = 7 WT neurons from 7 cultures) both for the apical and basal dendritic tree (Fig. 6B, C), being statistically significant in the apical part (at 120–140 µm from soma; p<0.05). An even stronger phenotype was found for granule cell-like ESNs (Fig. 6D). Knockout ESNs (n = 5 p75^NTR^-KO neurons from 3 cultures) turned out to be significantly more complex than the wild type (n = 8 WT neurons from 5 cultures; at 150–180 µm from the soma; p<0.05). The phenotype was seen in the more distally located part of the granule cell dendritic tree, suggesting that p75^NTR^-KO ESNs were longer, but also showed a more pronounced dendritic branching (Fig. 6E). Altogether, the analysis of p75^NTR^-KO ESNs is in line with the phenotype described before for mature pyramidal neurons in p75^NTR^-complete knock out mice [20].

**Figure 7:**
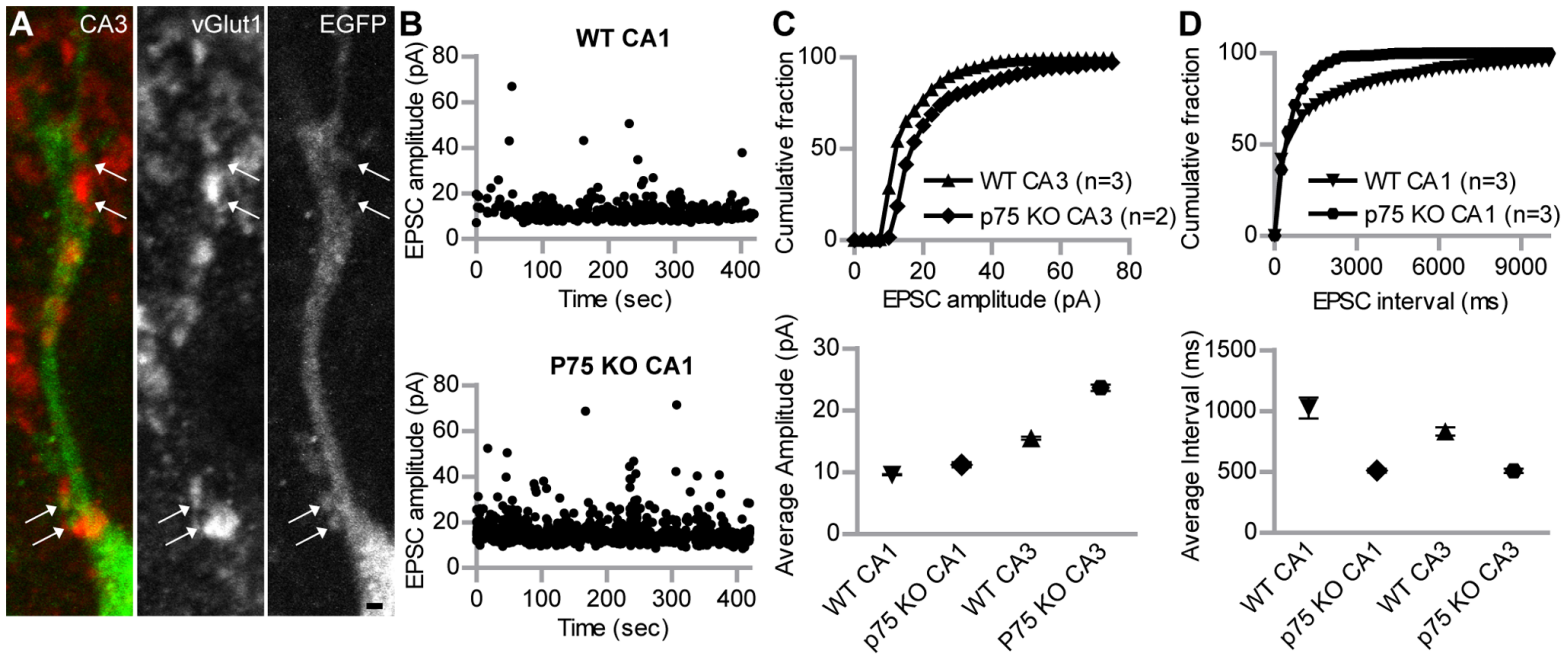
P75^NTR^-deficient ESNs show spine growth and functional maturation, but different EPSC properties than wild type. (A) Co-staining of p75^NTR^-deficient type A ESNs in the proximal apical compartment of CA3 ESNs (see grey box in figure 6 C); immunohistochemistry against EGFP and vGLUT1. Arrows point to co-localizations of dendritic spines with the pre-synaptic marker. (B) Representative recordings of mEPSC amplitudes of a wildtype ESN (top) and p75^NTR^-KO ESN in CA3 region (bottom), respectively. (C) Cumulative percentage of mEPSC amplitudes recorded in voltage-clamp recordings of WT ESNs and p75^NTR^-KO ESNs in CA3 region. The curve of p75^NTR^-KO ESNs reaches 100% later, indicating more mEPSCs with higher amplitude than in ESNs (top). In line with this, the total average amplitude of these cells is also higher (bottom). As the number of analyzed p75^NTR^-KO ESNs was only two and three, respectively, SEM was performed based on single EPSC events. WT CA1: n = 463; p75^NTR^-KO CA1: n = 579; WT CA3: n = 1110; p75^NTR^-KO CA3: n = 1232. (D) Cumulative percentage of mEPSC intervals of type A p75^NTR^-KO ESNs versus wild type ESNs recorded in voltage-clamp experiments. The curve of p75^NTR^-KO ESNs reaches 100% faster, indicating much shorter intervals between mEPSCs, compared to ESNs (top). The total average of mEPSC intervals shows a tendency to shorter intervals for both CA1 and CA3 p75^NTR^-KO ESNs (bottom). WT CA1: n = 893; p75^NTR^-KO CA1: n = 1028; WT CA3: n = 1773; p75^NTR^-KO CA3: n = 1157. Student’s t test, *p<0.05. Error bars represent SEM; Scale bar represents 1 µm.

Spine growth and corresponding co-localizing pre-synaptic structures were seen on CA3 type A p75^NTR^-KO ESNs (Fig. 7A), suggesting that p75^NTR^-KO ESNs receive excitatory synaptic input as seen for the WT ESNs before (Fig. 3). These findings were supported by mEPSC properties. P75^NTR^-deficient ESNs in the CA1 and CA3 region showed both higher averaged amplitudes (Fig. 7C, bottom) and a clear tendency in the cumulative fraction (Fig. 7C, top). The average interval between mEPSCs was smaller in both regions of p75^NTR^-KO ESNs in comparison to wild type ESNs (Fig. 7D bottom).

## Discussion

Using the differentiation of ES cells into a homogenous population of Pax6-positive ESNPs followed by a successful transplantation into OHCs we were able to show that ESNPs morphologically and functionally integrated into hippocampal tissue. The integrated and mature ESNs precisely adopted a hippocampal subfield-specific morphology of mature neurons. This referred to pyramidal-like cells which had been reported to derive from this kind of synthetic radial glia [21] as well as to granule-like neurons that are profoundly different in shape and size (Fig. 1D). Furthermore, immunohistochemical stainings of OHC cryosections show that subtype-specific markers for CA1 and granule neurons, respectively, are exclusively expressed by type A ESNs, but not by type B ESNs (Fig. S5).

Interestingly, typical morphological differences between intrinsic CA1 and CA3 pyramidal cells (Fig. 2B) were found to be significant for pyramidal-like CA1 and as well as CA3-ESNs (Fig. 2A). These data strongly suggest that local cues provided by the surrounding tissue influence dendritic arborization, according to the idea of extrinsic factors determining neuronal fate. However, previous transplantation studies using ESNPs produced via the same procedure report a restriction of their developmental plasticity. Transplanted into chick embryos, ESNs settled in the spinal cord elongated axons whereas those in the dorsal root ganglia did not [29]. In line with the known role of Pax6 and Emx2 during cortical development, Pax6-positive radial glia (RG) were only capable of acquiring a dorsal but not a ventral phenotype [30]. Additionally, a recent study using ES cell-derived motor neurons generated via a procedure based on intrinsic Wnt, Fgfs and Hh in the absence of retinoic acid, reports that ESNs acquire different motor pool identities [5]. The authors show that molecular markers anticipate functional properties and grafted ESNs settle in appropriate domains, irrespective of neighboring neurons. According to the data presented here, we hypothesize that the pre-determination of ESNPs achieved by the differentiation protocol [22] and intrinsic mechanisms narrows down the developmental potential to a glutamatergic cortical fate, but within this restriction maintains the responsiveness to external cues (like subfield-specific components such as cell adhesion molecules or secreted peptides) which results in the generation of different hippocampal subtypes. However in some cases, glia-like progeny of ESNPs was observed in dissociated growing cultures as well as in OHCs, where they did not show a preferred localization. The fact that these EGFP-positive cells were also positive for the glial fibrillary acidic protein (GFAP) (Fig. S3C) but negative for the microglial marker IB4 (Fig. S3D) suggests that a minor fraction of ESNPs adopted an astrocytic fate.

Previous transplantation studies into hippocampal tissue reported a functional integration in the sense that spikes and postsynaptic potentials could be detected. As our study, all of them were based on the integration into young neuronal tissue. Benninger et al. [12] who transplanted a heterogenous population of mouse ESNPs (produced via a different procedure) into the hilar region of rat hippocampal cultures did not investigate pyramidal cell morphologies. Interestingly, the *in vivo* transplantation approach of Wernig et al. [13] resulted in a heterogeneous outcome of pyramidal-like and immature-shaped ESNs irrespective of the brain region in which they were found. In a third study, transplantation of ESNPs (reported to be differentiated according to a similar protocol) into the neocortex *in vivo* led to pyramidal-like cells that carried rudimentary dendrites [31]. Thus, a complete structural integration of ESNs was not necessarily expected. Therefore, the high structural heterogeneity among ESNs revealed the need to distinguish between ESNs acquiring hippocampal neuron morphology and the remainder in order to point out the remarkable degree of maturation that was reached by a minor fraction of ESNs (Fig. 1D). Furthermore it can be excluded that non-integrated ESNs represented an immature stage of type A ESNs, since out of 50 ESNs that were imaged repeatedly, no type B ESN was observed to develop into a type A ESN. Conversely, the morphological maturation of a type A ESN was followed within a remarkably short time period (DIV 1–3) from an EGFP-negative progenitor (Fig. S6, for details see Materials and Methods S1). Regularly it was found that ESN cell bodies were interconnected. However, since the associated cells had neuronal identity (other than the fused microglia described in [32]) and fusion between neurons has not been reported, it is more likely that cytoplasmically connected ESNs originated from incomplete cell division rather than from cell fusion. The sequential tracking of a pyramidal type A ESN deriving from an EGFP-negative progenitor strengthens this hypothesis because fusion would implement the transfer of GFP protein from one cell to another which was not observed here (Fig. S6). Furthermore, the transplantation of ESNPs into OHCs prepared from transgenic mice expressing td-Tomato led to a type A ESN negative for td-Tomato, undermining the donor origin of the EGFP-expressing ESN (Fig. S7).

Adult neurogenesis, the subsequent integration of new neuronal components into the pre-existing network of the hippocampus is a highly regulated process involving active survival signaling of newly formed cells [33]. Accordingly, less than 30% of newly generated progenitors survive and become integrated into the mouse hippocampal circuitry [34]. Also in the study presented here we show that only a small fraction of transplanted cells adopted the shape of intrinsic neurons. While granule-like ESNs in the DG were as complex as intrinsic granule neurons (Fig. 1C) pyramidal-like ESNs did not reach the complexity of their intrinsic counterparts. The reason might be that due to the neurogenic niche [35], the DG provides an environment supporting integration.

In line with the dendritic fine morphology that revealed the presence of dendritic spines opposite pre-synaptic terminals (Fig. 3A, B), EPSCs could be detected on morphologically integrated (type A) ESNs (Fig. 3C). Intriguingly, type B ESNs neither received synaptic input nor did they develop the ability to fire action potentials (Fig. 3F, G). Therefore, type B ESNs were regarded immature and not properly integrated despite the synapsin-positive terminals that were found in close proximity to their spineless dendrites (Fig. 3E). The presence of ESNs that were not electrophysiologically active was surprising since it has been reported that morphologically region-related maturation is no prerequisite for functional integration [13]. Concentrating on type A ESNs we found that CA1 and CA3 ESNs presented qualities that lay within the range of intrinsic hippocampal neurons in a region-dependent manner. These results are in line with the view that type A ESNs successfully integrate in a functional manner into the local circuitry [12], [13]. However, using a homogenously pre-differentiated population of ESNPs, we provide a much more detailed description of region-specific morphological integration. We did not observe NMDA receptor-mediated currents in CA3 ESNs (Fig. 4D). This is in line with earlier reports [12], [13], but probably indeed a special feature of transplanted ESNs, since during endogenous adult neurogenesis functional NMDA receptors (with varying subunit compositions) are present at all neuronal maturation stages [36]. Furthermore, peak amplitude and frequency of action potentials specifically differed in CA1 but not in CA3 ESNs compared to controls (Fig. 5A-C). This indicates that CA1 ESNs are less mature since reduced AP amplitude has been linked to an immature stage before [12]. Similarly, a less negative membrane potential as well as an increased AP threshold have been reported during the course of maturation, the capacitance however is higher in our study (Table 1; see also [12]).

The neurotrophin receptor p75^NTR^ has been reported to be involved in multiple and partially growth-limiting processes during neuronal development. Highly expressed in an early stage, it was primarily shown to promote apoptosis but it also negatively controls dendritic complexity [20]. Most of the studies examining the function of p75^NTR^ were based on the constitutive knockout model. In order to circumvent possible compensatory mechanisms that could mask the phenotype we transplanted p75^NTR^-deficient neuronal progenitors into wild type OHCs. This approach enabled us to observe cell-autonomous effects of a lack of p75^NTR^, since single KO cells are placed into a WT environment. We did not see a difference in type A p75^NTR^-KO ESN outcome compared to WT ESNs, in contrast to another study which reported that in p75^NTR^-KO mice hippocampal neurogenesis was critically impaired [37]. Those animals from the Catts et al. study carried a targeted deletion of *exonIII*, leaving a pro-apoptotic fragment of p75^NTR^, whereas *exonIV* deletion, as used in the current study, results in a complete loss of p75^NTR^ [38]. Our morphological analysis revealed that ESNs lacking p75^NTR^ were more complex than wild type ESNs, in line with the known negatively modulating effect of p75^NTR^ in mature pyramidal neurons in the mouse KO model [20]. In the same study the overexpression of p75^NTR^ in single pyramidal neurons resulted in a decreased dendritic complexity in the proximal apical compartment, the exact region in which the lack of p75^NTR^ led to an increase in the current study (Fig. 6). This subcellular region might therefore be prone to cell-autonomous effects of p75^NTR^. In line with the antagonizing function of TrkB and p75^NTR^ is the observation that granule cells lacking the neurotrophin receptor TrkB were significantly less complex than the wild type [39]. Consistent with an increase of dendritic complexity in the proximal apical compartment of CA3 ESNs, these cells received increased synaptic input compared to wild type ESNs mainly through an increased frequency of EPSCs (Fig. 7C).

Taken together we were able to detect a cell-specific function of p75^NTR^ during maturation and integration of ES cell-derived neuronal precursors into the hippocampal network. This also proves that single labeled genetically-modified neurons can be observed over time as they integrate into the neuronal circuitry, examining structure and function in parallel. This approach can be specifically useful to unravel cell-autonomous effects of other proteins relevant in the context of synaptic plasticity and provides new means to study the developmental function of proteins that, if lacking in a conventional knockout, result in a lethal phenotype.

## Supporting Information

Figure S1 **Transplantation and survival in different hippocampal subfields.** (A) ESNPs are transplanted into DG, CA1 and CA3; CA4, EC (entorhinal cortex) and HF (hippocampal fissure) – other regions ESNs were detected (Fig. S1F). (B)-(D) DG, CA1 and CA3 regions, fixed two hours after transplantation and stained for EGFP (green) and DAPI (red). In the DG, 22 ESNPs were identified, in CA1 23, in CA3 18. (E) ESNs counted separately for slices transplanted into the respective regions; average ESN number at 32 DIV (DG: 6.4±0.9; CA1∶3.1±0.5; CA3∶3.5±0.6; n = 80 slices per transplanted region). (F) Percentage of cells detected in the regions of the slice culture (DG: 43.71±4.50; CA1∶12.56±1.87; CA3∶15.03±2.36; CA4∶0.46±0.31; EC: 1.76±1.47; HF: 16.51±3.49; P (periphery): 9.87±3.10) from 12 independent transplantations; n = 68 wells containing four slice cultures each. Error bars represent the standard error of the mean. Student’s t-test, *p<0.05. Scale bars 1 mm in A, 100 µm in B–D.
(PDF)

Figure S2 **Micrographs of ESNs within OHCs.** (A**)** Overview of EGFP-positive ESNs 14 days after transplantation into the dentate gyrus. (B-D) Micrographs and corresponding tracings of ESNs in different subfields of the hippocampus, in the DG (B), CA1 (C) and CA3 (D), respectively (see figure 1). Scale bars in A represent 1 mm; in B-D 100 µm
(PDF)

Figure S3 **Glia-like ESNP-derived cells resemble astrocytes.** (A) Glia-like EGFP-positive cell imaged live in the slice culture at 28 DIV. Its processes are in contact with thin EGFP-positive fibers, presumably ESN axons (arrows). (B) Dissociated growing ESN culture at 15 DIV. GFAP (glial fibrillary acidic protein) expression is shown in red. (C) Higher magnification view of boxed region in B. Arrows point to association sites of GFAP-positive glial processes with GFAP-negative EGFP-positive fibers. The cell body of the glial cell shows a faint EGFP staining (arrowhead). (D) EGFP-positive glia in OHCs (arrows) are IB4-negative, intrinsic microglia (arrowheads) do not express EGFP. Scale bars in A and C 10 µm; in B and D 100 µm.
(PDF)

Figure S4 **Micrographs of p75-KO ESNs within OHCs.** Micrographs and corresponding tracings of ESNs in the CA3 (A) and the DG (B) of the hippocampus (see figure 6). Scale bars 100 µm.
(PDF)

Figure S5 **ESNs partially adopt the immunohistochemical identity of resident hippocampal neurons.** (A) Type B DG ESN is Ctip2-negative (arrow). Arrowhead points to Ctip2-positive intrinsic granule neuron. (B) Type A CA1 ESN shows positive Ctip2 immunoreactivity (arrow) like intrinsic CA1 pyramidal neurons (arrowhead). (C) Type B DG ESN is Prox1-negative (arrow); in the same section, Prox1-positive intrinsic granule neurons were seen (arrowhead). (D) DG ESN associated to the cell in C. It shows a weak Prox1 signal (arrow). Cryosections were made 32 days post transplantation; Scale bars 10 µm.
(PDF)

Figure S6 **Maturation into a pyramidal-like cell occurs within 4 days.** Live imaging time course 1–4 days after transplantation of ESNPs into hippocampal slice culture, CA3 region. Top row – tracings of the same neuron, the axon is shown in red. Dots indicate that some dendrites were not completely imaged. Bottom row – sections of live images at the respective DIV. Increasing fluorescence intensity is clearly visible over time. Scale bar 100 µm.
(PDF)

Figure S7 **ESNP transplanted into OHC from Tomato expressing transgenic mice (1) is of donor origin.** (A) Maximum intensity projection of type A ESN in the CA3 region. (B-D) Higher magnification view of boxed regions of dendrites (B, C) and soma (D) in A. Arrows point to EGFP-positive parts of the ESN that do not show red fluorescence. Neighboring dendrites of host neurons, however, are Tomoto (arrowheads). Scale bars represent 10 µm.
(PDF)

Materials and Methods S1 (DOCX)
