# Correction: Region-Specific Integration of Embryonic Stem Cell-Derived Neuronal Precursors into a Pre-Existing Neuronal Circuit

**DOI:** 10.1371/annotation/d4eaf996-d270-4270-a186-36c92ec1066c

**Published:** 2014-01-02

**Authors:** Franziska Neuser, Martin Polack, Christine Annaheim, Kerry L. Tucker, Martin Korte

The supporting information files are duplicates of the figure files. Please see the correct supporting information files here:

Figure S1: 

Figure S2: 

Figure S3: 

Figure S4: 

Figure S5: 

Figure S6: 

Figure S7: 
