# Supplementary figures and images for: Region-Specific Integration of Embryonic Stem Cell-Derived Neuronal Precursors into a Pre-Existing Neuronal Circuit
Source: PLoS One. 2013 Jun 20;8(6):e66497. doi: 10.1371/journal.pone.0066497 (PMC3688776; doi:10.1371/journal.pone.0066497)

**Figure 1**

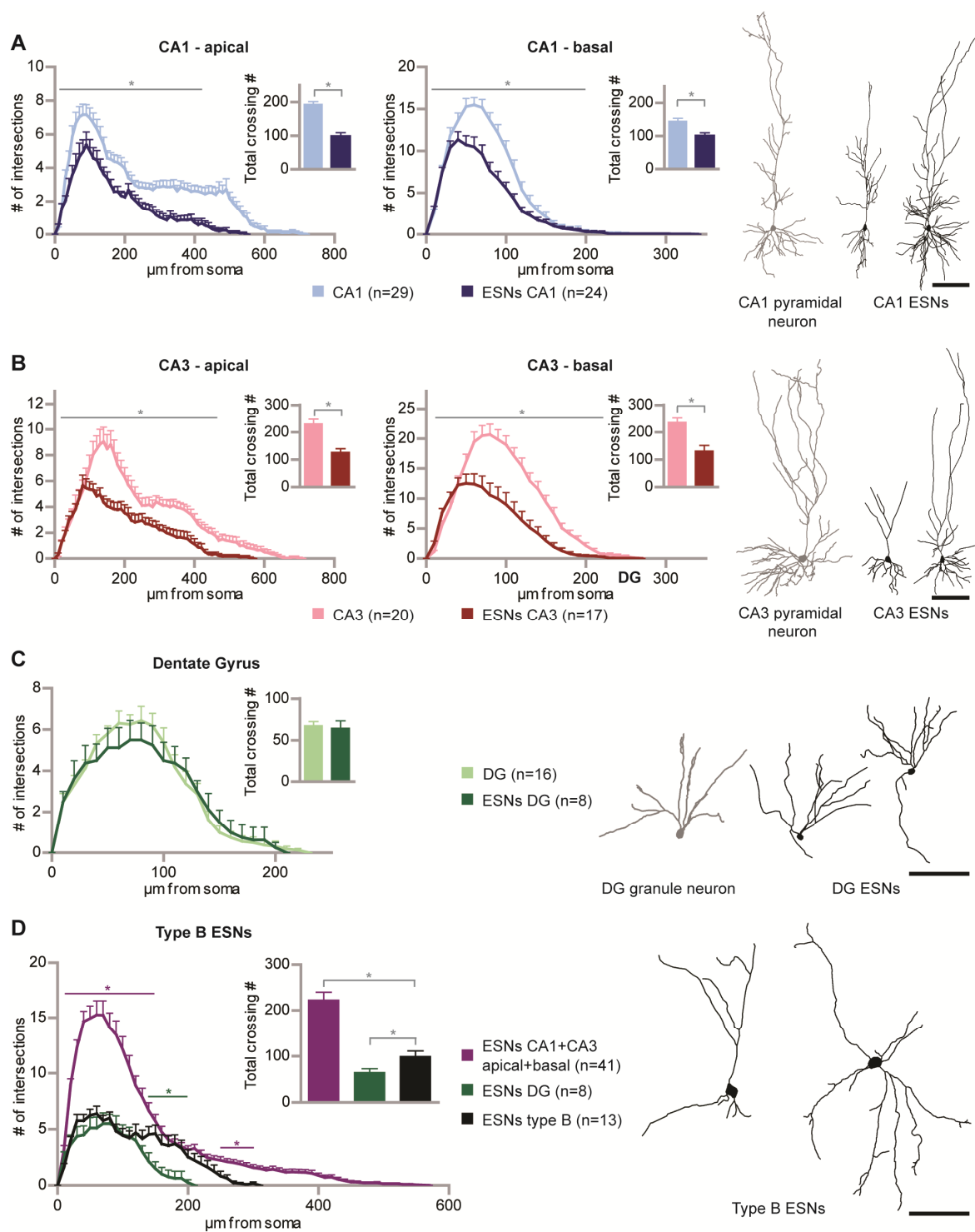

Supplement: Figure S1 — Transplantation and survival in different hippocampal subfields. (A) ESNPs are transplanted into DG, CA1 and CA3; CA4, EC (entorhinal cortex) and HF (hippocampal fissure) – other regions ESNs were detected (Fig. S1F). (B)-(D) DG, CA1 and CA3 regions, fixed two hours after transplantation and stained for EGFP (green) and DAPI (red). In the DG, 22 ESNPs were identified, in CA1 23, in CA3 18. (E) ESNs counted separately for slices transplanted into the respective regions; average ESN number at 32 DIV (DG: 6.4±0.9; CA1∶3.1±0.5; CA3∶3.5±0.6; n = 80 slices per transplanted region). (F) Percentage of cells detected in the regions of the slice culture (DG: 43.71±4.50; CA1∶12.56±1.87; CA3∶15.03±2.36; CA4∶0.46±0.31; EC: 1.76±1.47; HF: 16.51±3.49; P (periphery): 9.87±3.10) from 12 independent transplantations; n = 68 wells containing four slice cultures each. Error bars represent the standard error of the mean. Student’s t-test, *p<0.05. Scale bars 1 mm in A, 100 µm in B–D. (PDF) [file pone.0066497.s001.pdf]

**Figure 2**

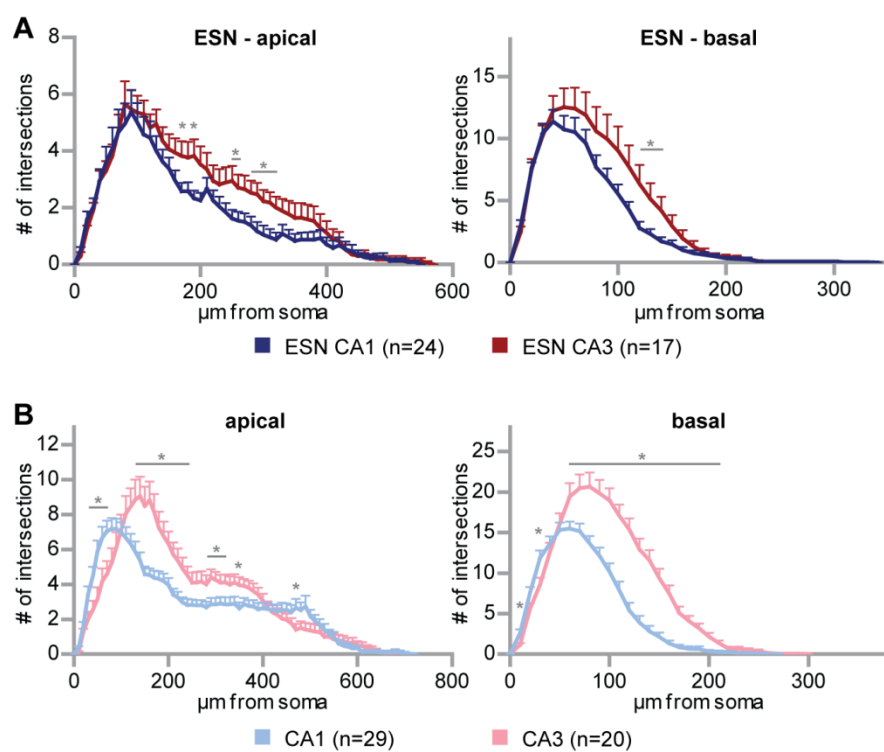

Supplement: Figure S2 — Micrographs of ESNs within OHCs. (A) Overview of EGFP-positive ESNs 14 days after transplantation into the dentate gyrus. (B-D) Micrographs and corresponding tracings of ESNs in different subfields of the hippocampus, in the DG (B), CA1 (C) and CA3 (D), respectively (see figure 1). Scale bars in A represent 1 mm; in B-D 100 µm (PDF) [file pone.0066497.s002.pdf]

**Figure 3**

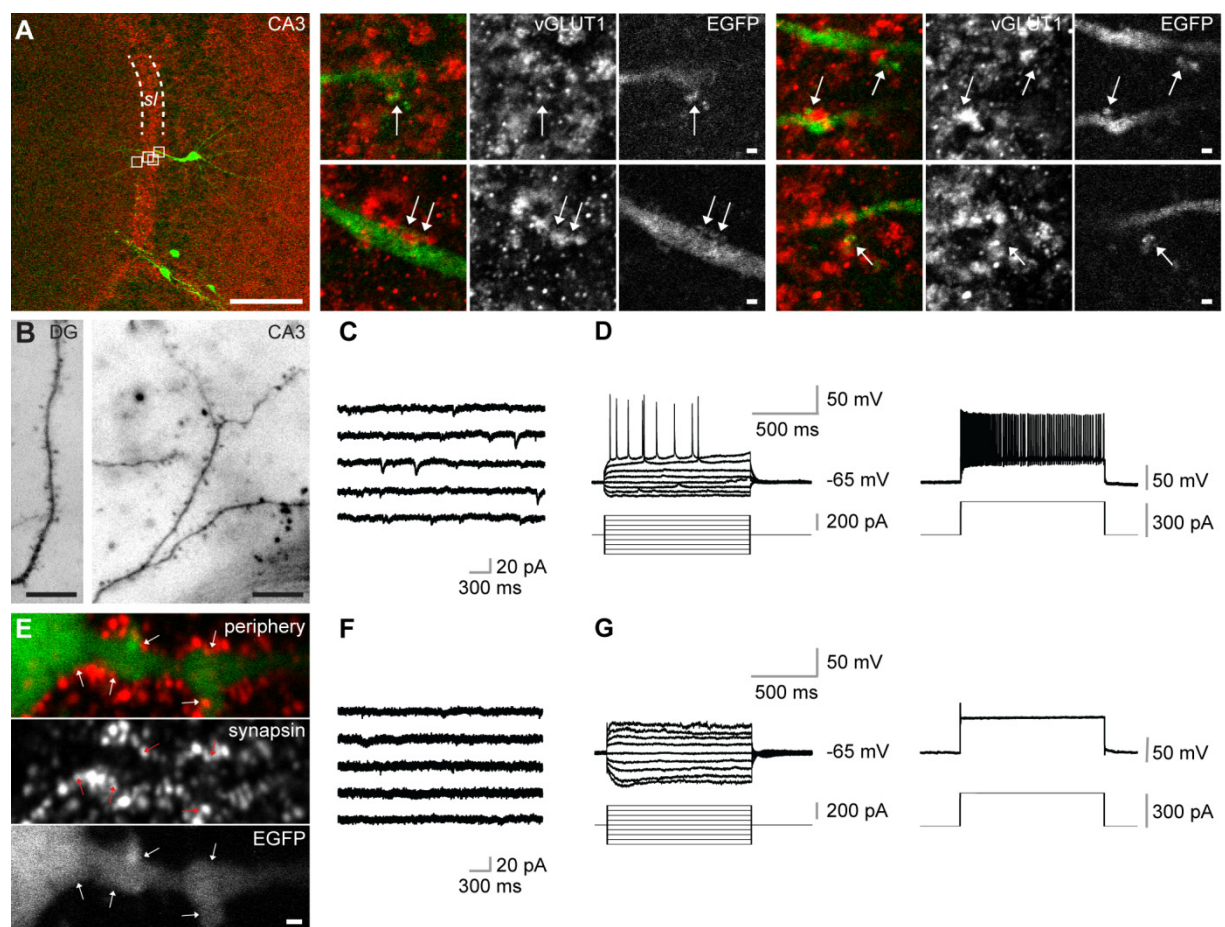

Supplement: Figure S3 — Glia-like ESNP-derived cells resemble astrocytes. (A) Glia-like EGFP-positive cell imaged live in the slice culture at 28 DIV. Its processes are in contact with thin EGFP-positive fibers, presumably ESN axons (arrows). (B) Dissociated growing ESN culture at 15 DIV. GFAP (glial fibrillary acidic protein) expression is shown in red. (C) Higher magnification view of boxed region in B. Arrows point to association sites of GFAP-positive glial processes with GFAP-negative EGFP-positive fibers. The cell body of the glial cell shows a faint EGFP staining (arrowhead). (D) EGFP-positive glia in OHCs (arrows) are IB4-negative, intrinsic microglia (arrowheads) do not express EGFP. Scale bars in A and C 10 µm; in B and D 100 µm. (PDF) [file pone.0066497.s003.pdf]

**Figure 4**

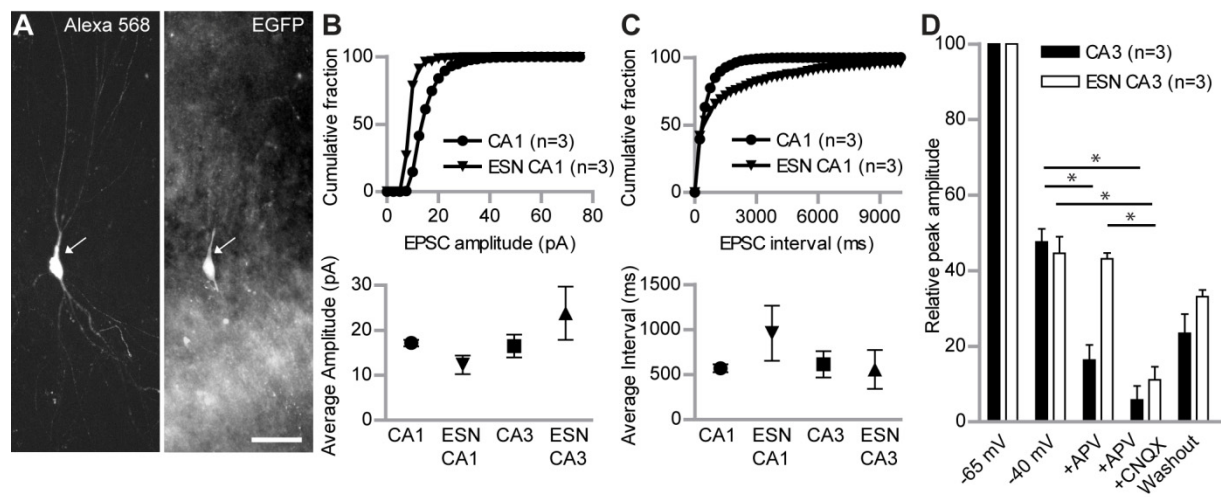

Supplement: Figure S4 — Micrographs of p75-KO ESNs within OHCs. Micrographs and corresponding tracings of ESNs in the CA3 (A) and the DG (B) of the hippocampus (see figure 6). Scale bars 100 µm. (PDF) [file pone.0066497.s004.pdf]

Figure 5

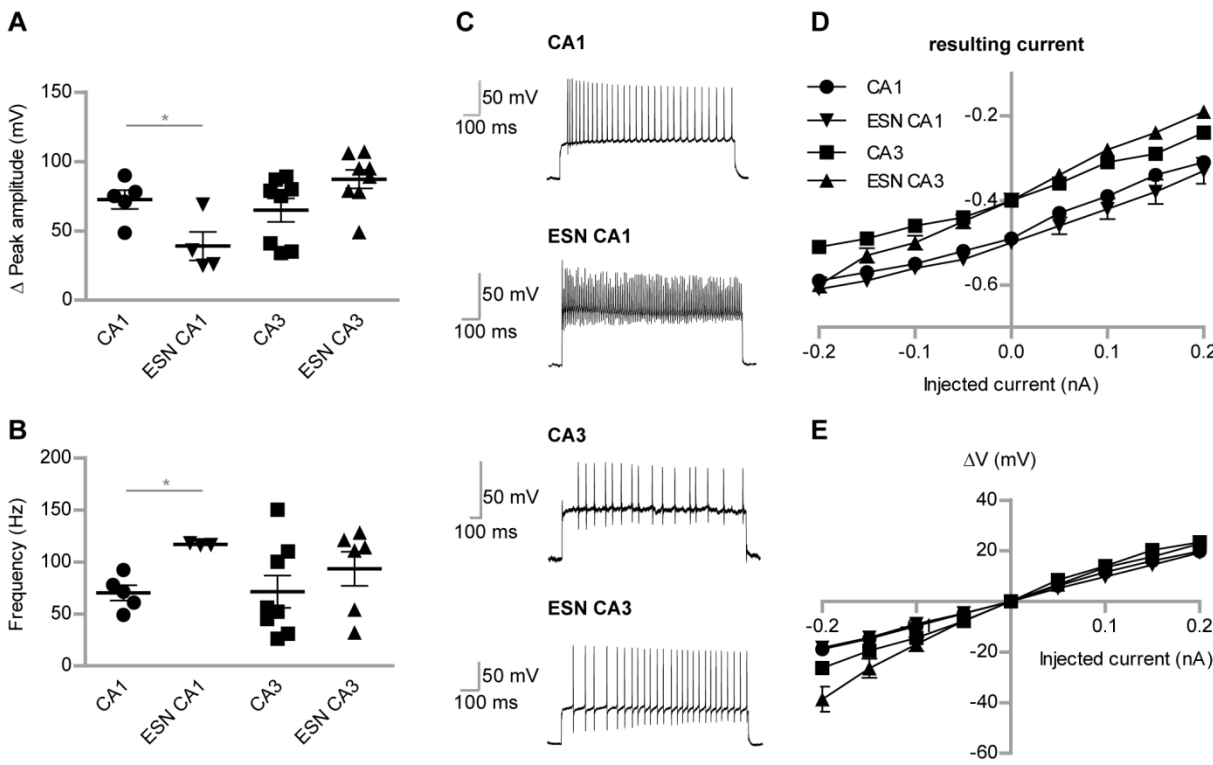

Supplement: Figure S5 — ESNs partially adopt the immunohistochemical identity of resident hippocampal neurons. (A) Type B DG ESN is Ctip2-negative (arrow). Arrowhead points to Ctip2-positive intrinsic granule neuron. (B) Type A CA1 ESN shows positive Ctip2 immunoreactivity (arrow) like intrinsic CA1 pyramidal neurons (arrowhead). (C) Type B DG ESN is Prox1-negative (arrow); in the same section, Prox1-positive intrinsic granule neurons were seen (arrowhead). (D) DG ESN associated to the cell in C. It shows a weak Prox1 signal (arrow). Cryosections were made 32 days post transplantation; Scale bars 10 µm. (PDF) [file pone.0066497.s005.pdf]

**Figure 6**

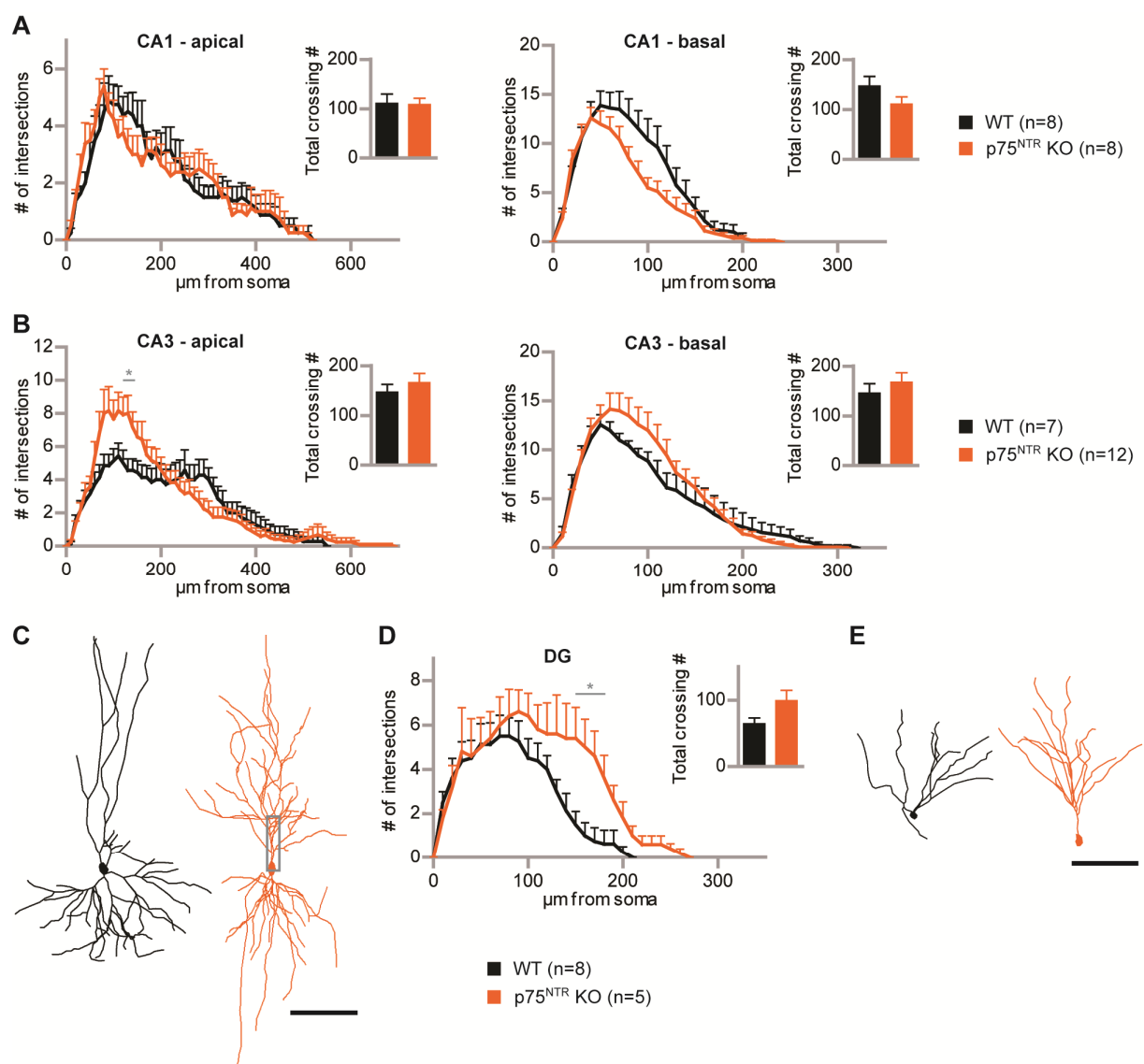

Supplement: Figure S6 — Maturation into a pyramidal-like cell occurs within 4 days. Live imaging time course 1–4 days after transplantation of ESNPs into hippocampal slice culture, CA3 region. Top row – tracings of the same neuron, the axon is shown in red. Dots indicate that some dendrites were not completely imaged. Bottom row – sections of live images at the respective DIV. Increasing fluorescence intensity is clearly visible over time. Scale bar 100 µm. (PDF) [file pone.0066497.s006.pdf]

**Figure S7**

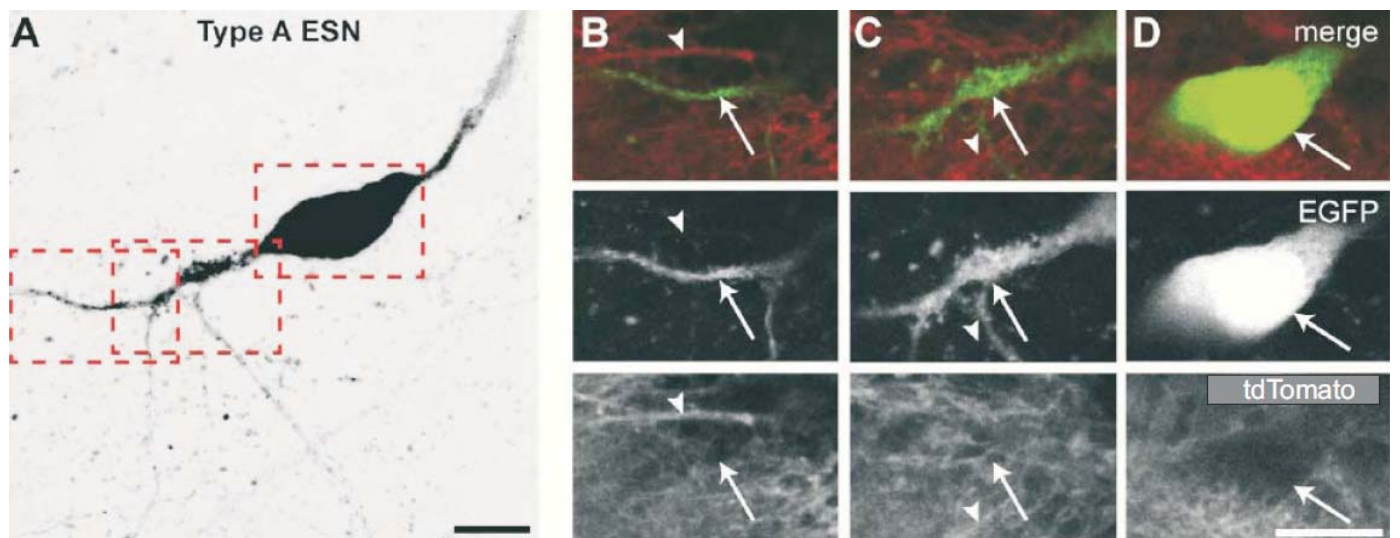

Supplement: Figure S7 — ESNP transplanted into OHC from Tomato expressing transgenic mice (1) is of donor origin. (A) Maximum intensity projection of type A ESN in the CA3 region. (B-D) Higher magnification view of boxed regions of dendrites (B, C) and soma (D) in A. Arrows point to EGFP-positive parts of the ESN that do not show red fluorescence. Neighboring dendrites of host neurons, however, are Tomoto (arrowheads). Scale bars represent 10 µm. (PDF) [file pone.0066497.s007.pdf]
