# Supplementary material for: Region-Specific Integration of Embryonic Stem Cell-Derived Neuronal Precursors into a Pre-Existing Neuronal Circuit
Source: PLoS One. 2013 Jun 20;8(6):e66497. doi: 10.1371/journal.pone.0066497 (PMC3688776; doi:10.1371/journal.pone.0066497)
Supplement: Materials and Methods S1 — (DOCX) [file pone.0066497.s008.docx]

**Supporting material methods:**

Cryosections of OHCs: Immunohistochemistry of OHC resulted in a superficial staining. Therefore cryosections of OHCs were used to detect hippocampal subtype-specific markers. After fixation and washing as described above, OHCs were carefully detached from the membrane. They were dehydrated in 30% sucrose over night at 4°C and subsequently frozen onto a piece of parafilm in a drop of freezing tissue Tek® (Sakura, Netherlands). Cryosections (30 µm) were prepared using a freezing microtome and immunohistochemistry was performed in a free-floating state as described above (blocking 1h, primary antibody over night, secondary antibody 2h). Finally, sections were DAPI-stained (1:1000 in PBS) and mounted in Fluoro-Gel®.

The transgenic mice used for the test of fusion of endogenous neurons with GFP-expressing ESNs (Figure S6), expressed the membrane-targeted tandemdimer Tomato (mT) ubiquitously, for details see (1) under supporting literature.

**Supporting literature:**

1. Muzumdar MD, Tasic B, Miyamichi K, Li L, Luo L (2007) A global double-fluorescent Cre reporter mouse. Genesis 45: 593-605. 10.1002/dvg.20335 [doi].
