# Supplementary figures and images for: Correction: Region-Specific Integration of Embryonic Stem Cell-Derived Neuronal Precursors into a Pre-Existing Neuronal Circuit
Source: PLoS One. 2014 Jan 2;9(1):10.1371/annotation/d4eaf996-d270-4270-a186-36c92ec1066c. doi: 10.1371/annotation/d4eaf996-d270-4270-a186-36c92ec1066c (PMC3887150; doi:10.1371/annotation/d4eaf996-d270-4270-a186-36c92ec1066c)

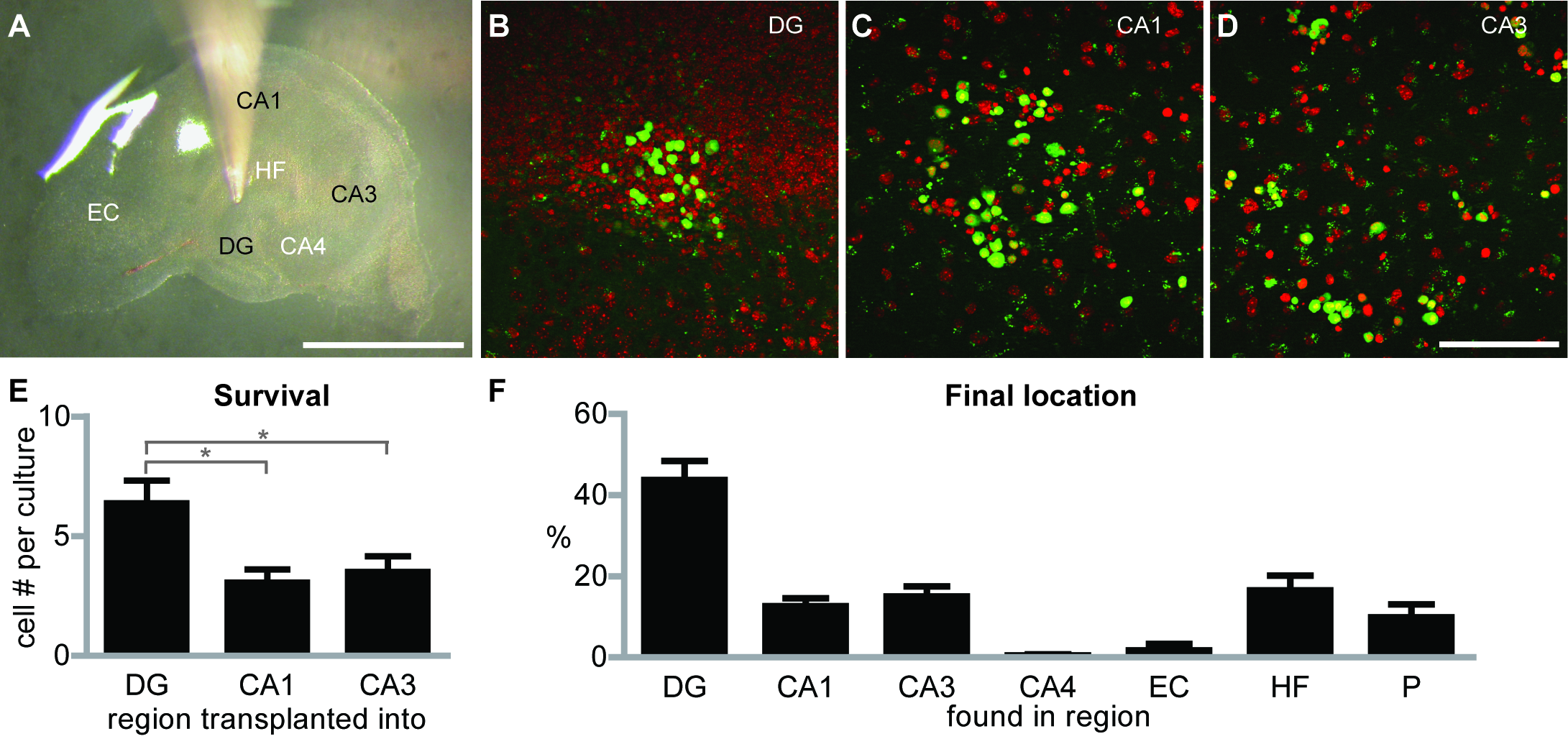

Supplement: Supplementary file 1 [file pone.d4eaf996-d270-4270-a186-36c92ec1066c.s001.tif]

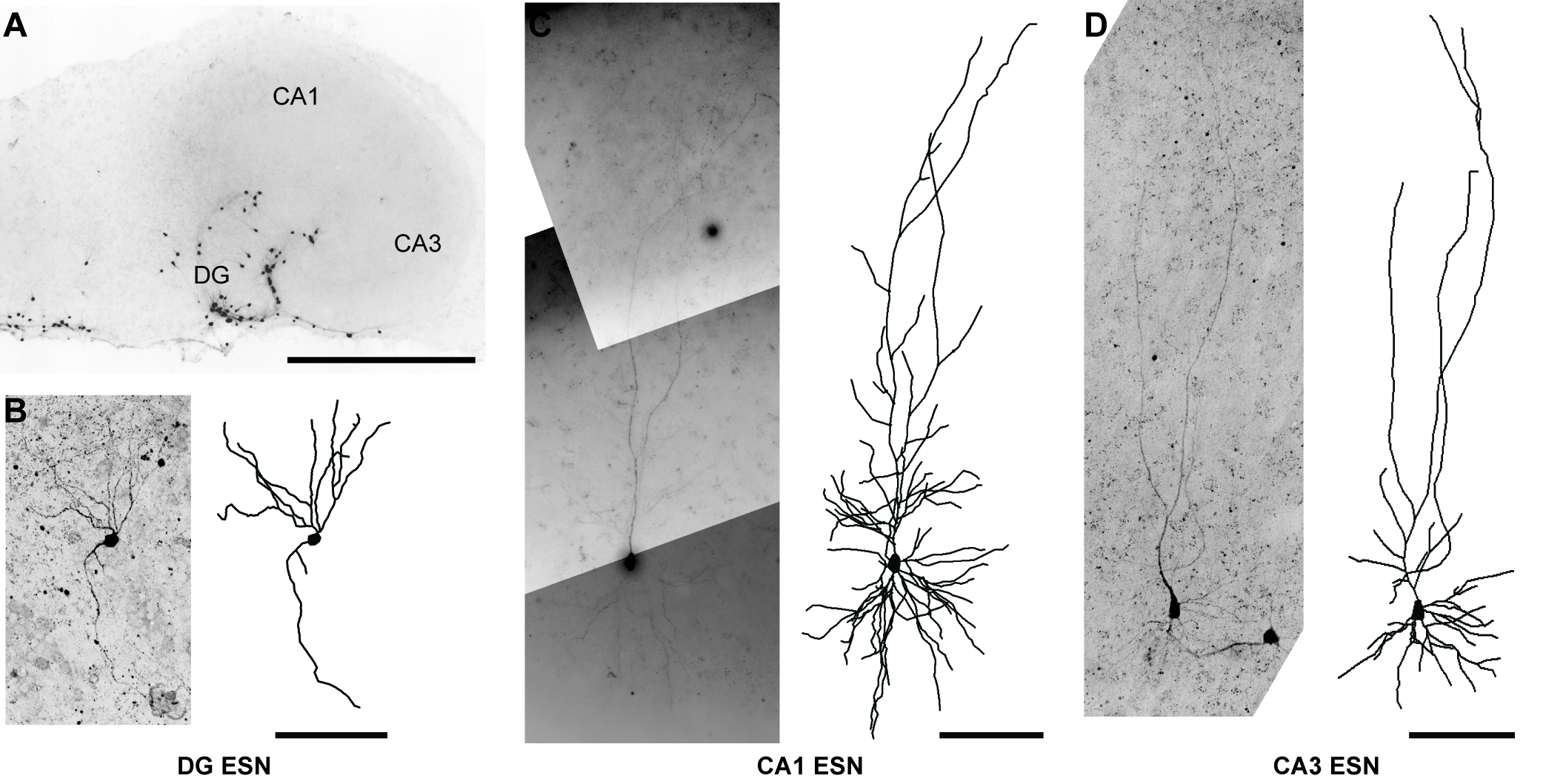

Supplement: Supplementary file 2 [file pone.d4eaf996-d270-4270-a186-36c92ec1066c.s002.tif]

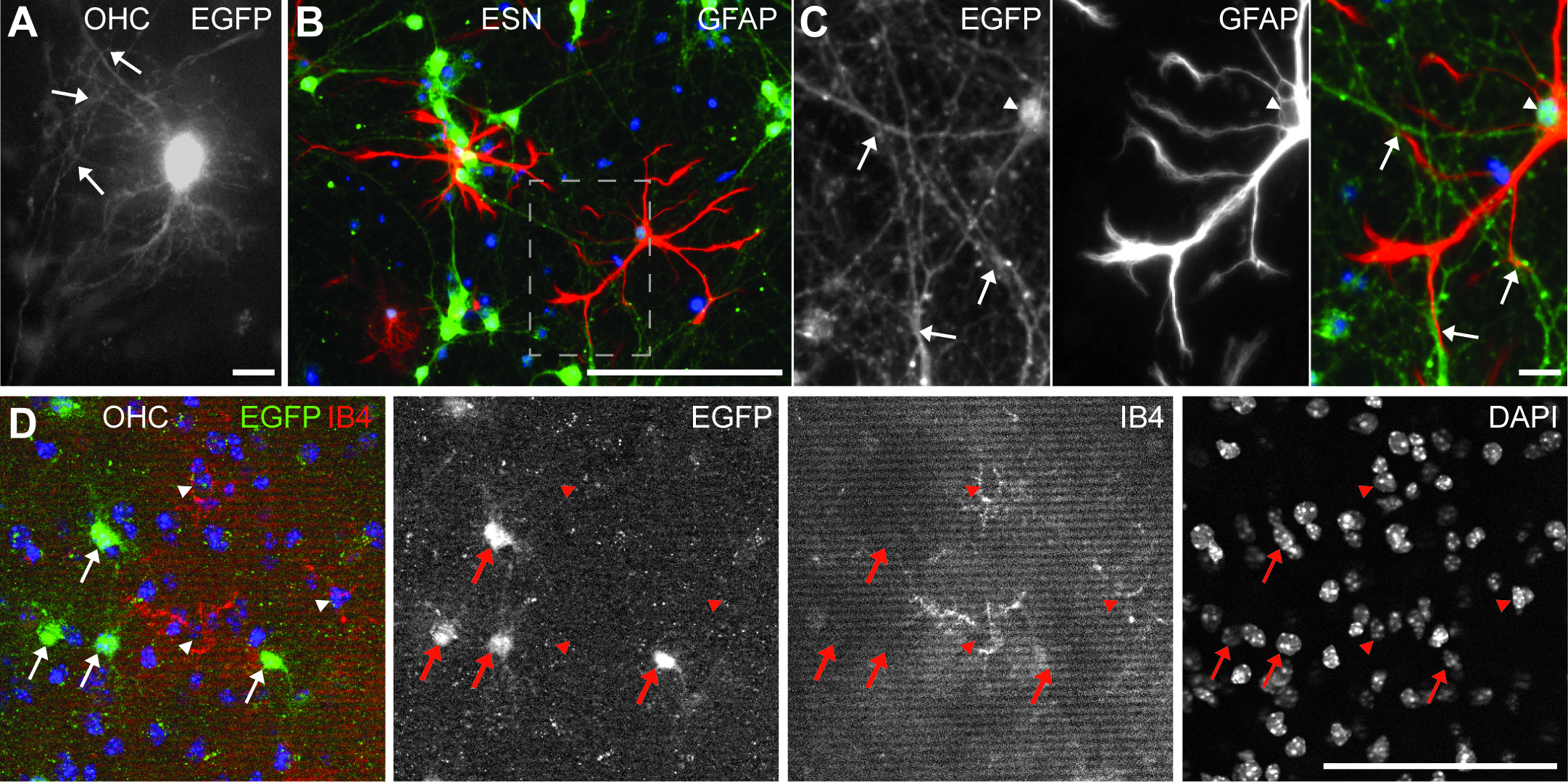

Supplement: Supplementary file 3 [file pone.d4eaf996-d270-4270-a186-36c92ec1066c.s003.tif]

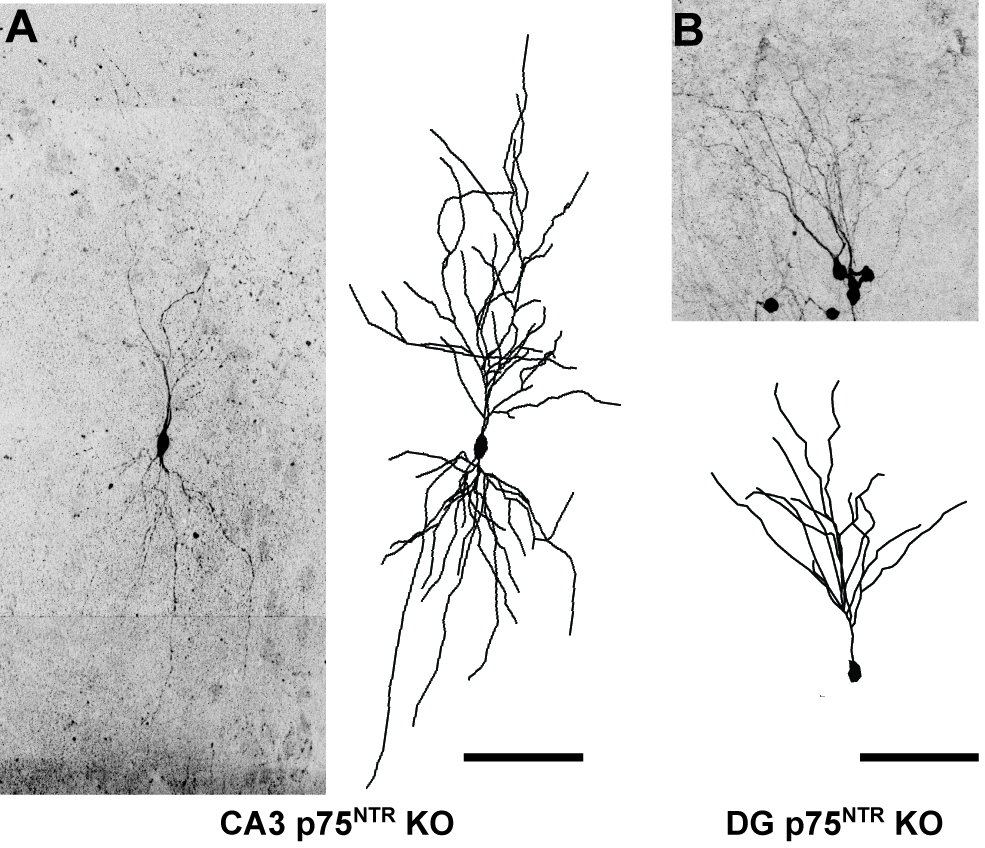

Supplement: Supplementary file 4 [file pone.d4eaf996-d270-4270-a186-36c92ec1066c.s004.tif]

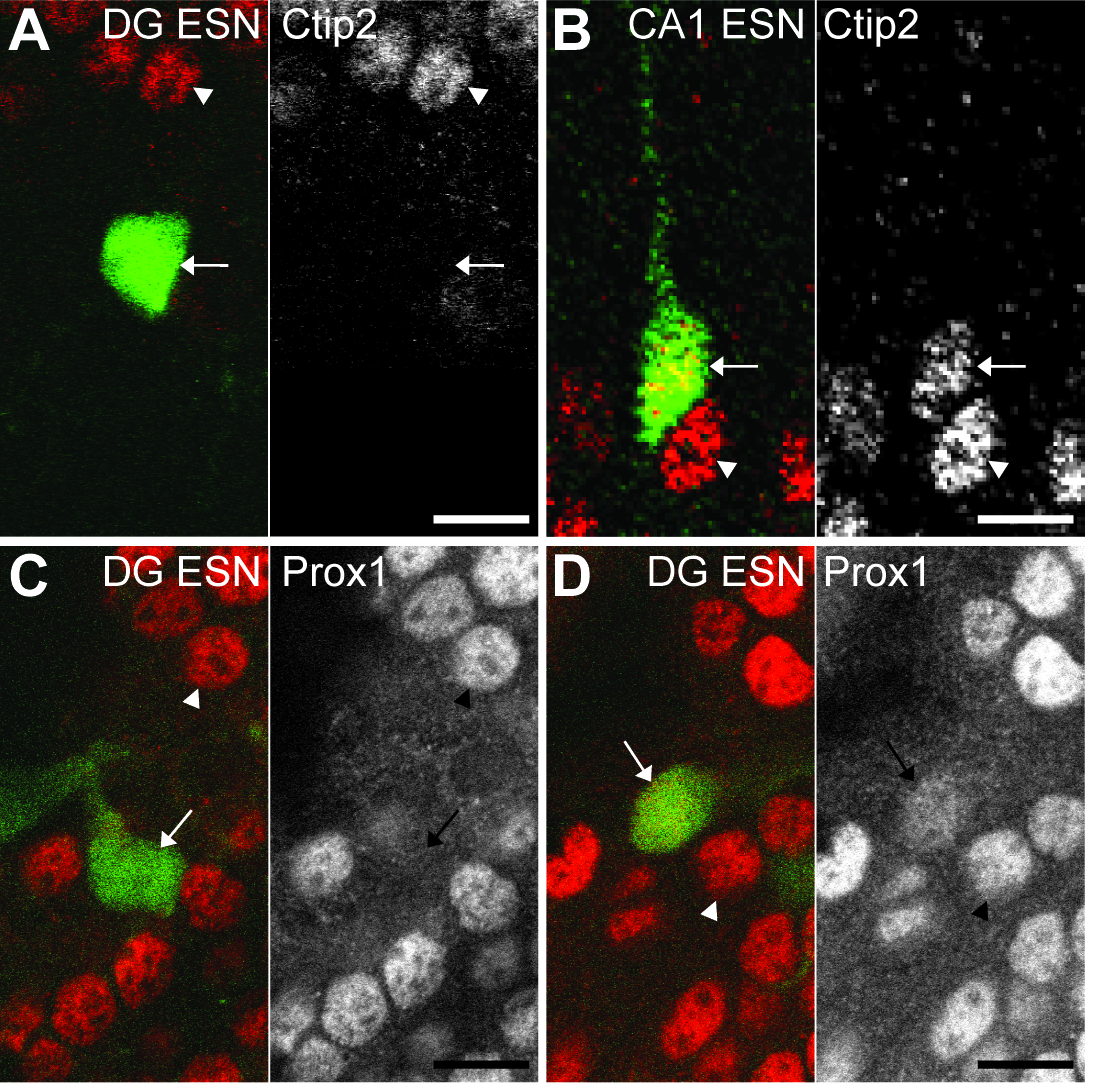

Supplement: Supplementary file 5 [file pone.d4eaf996-d270-4270-a186-36c92ec1066c.s005.tif]

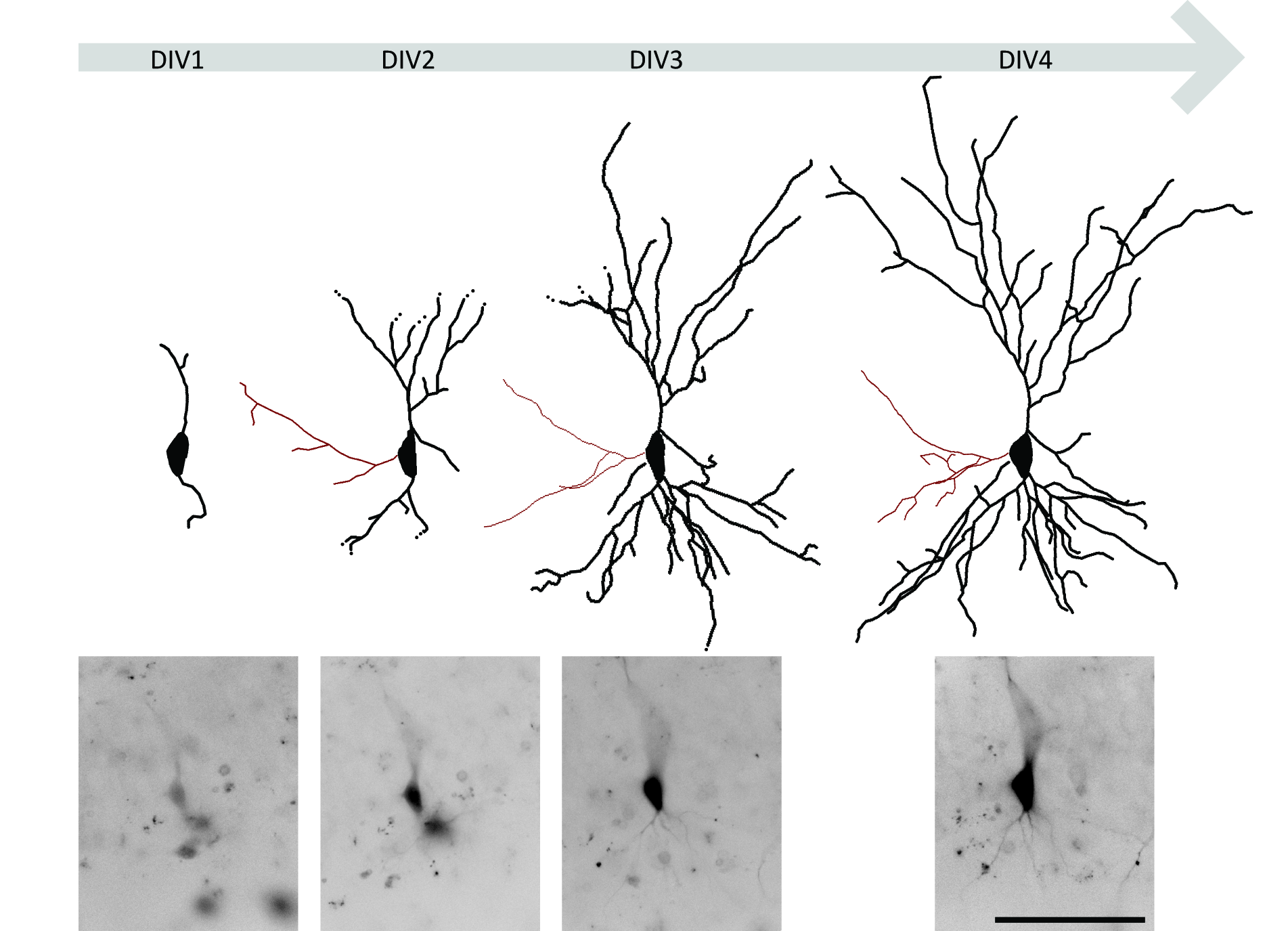

Supplement: Supplementary file 6 [file pone.d4eaf996-d270-4270-a186-36c92ec1066c.s006.tif]

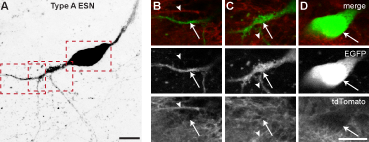

Supplement: Supplementary file 7 [file pone.d4eaf996-d270-4270-a186-36c92ec1066c.s007.tif]
